# Supplementary material for: Mycobacterium vaccae NCTC 11659, a Soil-Derived Bacterium with Stress Resilience Properties, Modulates the Proinflammatory Effects of LPS in Macrophages
Source: Int J Mol Sci. 2023 Mar 8;24(6):5176. doi: 10.3390/ijms24065176 (PMC10049321; doi:10.3390/ijms24065176)
Supplement: Supplementary file 1 [file ijms-24-05176-s001.zip › Ratio calculation example_101222_EH.pdf]

In the attached Excel file, there are 3 sheets: “Example IL10”, “Example IL12B”, and “Ratio examples”.

In the sheets “Example IL10” and “Example IL12B”, relative expression values are generated using the popular $2^{-\Delta\Delta Cq}$ method.

In the sheet “Ratio examples” the IL10/IL12B ratio is calculated 2 ways. The first method divides the relative expression values calculated from the $2^{-\Delta\Delta Cq}$ method, which isn’t very informative. What this calculation tells us isn’t the ratio of IL10/IL12B gene expression, but instead gives us the ratio of the relative IL10 expression (relative to the lowest gene expression in the IL10 sheet) to the relative IL12B expression (again, relative to the lowest expression in the IL12B sheet)—very confusing.

In the “Ratio examples” sheet, please see row 8 highlighted in red. The IL10/IL12B ratio is 1 despite the IL10 Cq being 20.5 and the IL12B Cq being 29.6 highlighted in green (remembering lower Cq values mean more gene expression, with a Cq difference of 1 representing a 2-fold change and a Cq difference of 2 representing a 4-fold change). This value of 1 doesn’t represent the value that we want, which is the direct ratio of IL10 to IL12B.

Method 2 is the method we use in the Lowry lab. Simply, the delta value is found between IL10 and IL12B (which is the difference in Cq values between the 2 genes) and this delta value is finally transformed into a fold change/relative expression value. Essentially, we skip the step in the $2^{-\Delta\Delta Cq}$ method that sets the relative expression value of the gene with the lowest Cq to 1 so we can get a direct ratio of IL10 to IL12B.

In Method 2, you might notice there is no normalization step with a housekeeping gene. This isn’t necessary, as we run genes using singleplex PCR assays running 1 gene at a time and only measuring the housekeeping gene once. Thus, if one were to modify Method 2 to include a normalization step, in our circumstance both methods would have the same outcome.
