# Supplementary material for: Mycobacterium vaccae NCTC 11659, a Soil-Derived Bacterium with Stress Resilience Properties, Modulates the Proinflammatory Effects of LPS in Macrophages
Source: Int J Mol Sci. 2023 Mar 8;24(6):5176. doi: 10.3390/ijms24065176 (PMC10049321; doi:10.3390/ijms24065176)
Supplement: Supplementary file 1 [file ijms-24-05176-s001.zip › Supplemental S1.qPCR THP-1 supplementary materials_proofs_022123_EH_CAL_AAC2.pdf]

S1. List of abbreviations for all gene symbols

AGER, advanced glycosylation end-product specific receptor
ALOX5, arachidonate 5-lipoxygenase
AREG, amphiregulin
ATF2, activating transcription factor 2
BCL2L1, BCL2 like 1
BCL6, BCL6 transcription repressor
BIRC2, baculoviral IAP repeat containing 2
C1QA, complement C1q A chain
C1QB, complement C1q B chain
C1S, complement C1s
C2, complement C2
C3, complement C3
C3AR1, complement C3a receptor 1
C4A, complement C4A (Rodgers blood group)
C5, complement C5
CCL13, C-C motif chemokine ligand 13
CCL17, C-C motif chemokine ligand 17
CCL19, C-C motif chemokine ligand 19
CCL2, C-C motif chemokine ligand 2
CCL20, C-C motif chemokine ligand 20
CCL21, C-C motif chemokine ligand 21
CCL22, C-C motif chemokine ligand 22
CCL23, C-C motif chemokine ligand 23
CCL24, C-C motif chemokine ligand 24
CCL3, C-C motif chemokine ligand 3
CCL4, C-C motif chemokine ligand 4
CCL5, C-C motif chemokine ligand 5
CCL8, C-C motif chemokine ligand 8
CCR1, C-C motif chemokine receptor 1
CCR2, C-C motif chemokine receptor 2
CCR7, C-C motif chemokine receptor 7
CD163, CD163 molecule
CD4, CD4 molecule
CD40, CD40 molecule
CD86, CD86 molecule
CEBPB, CCAAT enhancer binding protein beta
CFB, complement factor B
CFD, complement factor D
CFL1, cofilin 1
CREB1, cAMP responsive element binding protein 1
CSF1, colony stimulating factor 1
CSF3, colony stimulating factor 3
CXCL1, C-X-C motif chemokine ligand 1
CXCL10, C-X-C motif chemokine ligand 10

CXCL2, C-X-C motif chemokine ligand 2
CXCL3, C-X-C motif chemokine ligand 3
CXCL5, C-X-C motif chemokine ligand 5
CXCL6, C-X-C motif chemokine ligand 6
CXCR2, C-X-C motif chemokine receptor 2
CXCR4, C-X-C motif chemokine receptor 4
CYSLTR1, cysteinyl leukotriene receptor 1
DAXX, death domain associated protein
DDIT3, DNA damage inducible transcript 3
DEFA1, defensin alpha 1
ELK1, ETS transcription factor ELK1
FOS, Fos proto-oncogene, AP-1 transcription factor subunit
FXYD2, FXYD domain containing ion transport regulator 2
GNAQ, G protein subunit alpha q
GNAS, GNAS complex locus
GRB2, growth factor receptor bound protein 2
HDAC4, histone deacetylase 4
HIF1A, hypoxia inducible factor 1 subunit alpha
HLA-DRA, major histocompatibility complex, class II, DR alpha
HLA-DRB1, major histocompatibility complex, class II, DR beta 1
HMGB1, high mobility group box 1
HMGB2, high mobility group box 2
HMGN1, high mobility group nucleosome binding domain 1
HRAS, HRas proto-oncogene, GTPase
HSPB1, heat shock protein family B (small) member 1
IFI44, interferon induced protein 44
IFIT1, interferon induced protein with tetratricopeptide repeats 1
IFIT2, interferon induced protein with tetratricopeptide repeats 2
IFIT3, interferon induced protein with tetratricopeptide repeats 3
IL10, interleukin 10
IL10RB, interleukin 10 receptor subunit beta
IL11, interleukin 11
IL12B, interleukin 12B
IL15, interleukin 15
IL18, interleukin 18
IL1A, interleukin 1 alpha
IL1B, interleukin 1 beta
IL1R1, interleukin 1 receptor type 1
IL1RAP, interleukin 1 receptor accessory protein
IL1RN, interleukin 1 receptor antagonist
IL23A, interleukin 23 subunit alpha
IL23R, interleukin 23 receptor
IL6R, interleukin 6 receptor
IL8, C-X-C motif chemokine ligand 8
IRF1, interferon regulatory factor 1
IRF3, interferon regulatory factor 3

IRF5, interferon regulatory factor 5
IRF7, interferon regulatory factor 7
ITGB2, integrin subunit beta 2
KEAP1, kelch like ECH associated protein 1
LIMK1, LIM domain kinase 1
LTB, lymphotoxin beta
LTB4R, leukotriene B4 receptor
LTB4R2, leukotriene B4 receptor 2
LY96, lymphocyte antigen 96
MAFF, MAF bZIP transcription factor F
MAFG , MAF bZIP transcription factor G
MAFK , MAF bZIP transcription factor K
MAP2K1, mitogen-activated protein kinase kinase 1
MAP2K4, mitogen-activated protein kinase kinase 4
MAP2K6, mitogen-activated protein kinase kinase 6
MAP3K1, mitogen-activated protein kinase kinase kinase 1
MAP3K5, mitogen-activated protein kinase kinase kinase 5
MAP3K7, mitogen-activated protein kinase kinase kinase 7
MAPK1, mitogen-activated protein kinase 1
MAPK14, mitogen-activated protein kinase 14
MAPK3, mitogen-activated protein kinase 3
MAPK8, mitogen-activated protein kinase 8
MAPKAPK2, MAPK activated protein kinase 2
MAPKAPK5, MAPK activated protein kinase 5
MAX, MYC associated factor X
MEF2C, myocyte enhancer factor 2C
MEF2D, myocyte enhancer factor 2D
MKNK1, MAPK interacting serine/threonine kinase 1
MMP9, matrix metallopeptidase 9
MRC1, mannose receptor C-type 1
MX1, MX dynamin like GTPase 1
MX2, MX dynamin like GTPase 2
MYC, MYC proto-oncogene, bHLH transcription factor
MYD88, MYD88 innate immune signal transduction adaptor
NFATC3, nuclear factor of activated T cells 3
NFE2L2, nuclear factor, erythroid 2 like 2
NFKB1, nuclear factor kappa B subunit 1
NLRP3, NLR family pyrin domain containing 3
NOD1, nucleotide binding oligomerization domain containing 1
NOD2, nucleotide binding oligomerization domain containing 2
NR3C1, nuclear receptor subfamily 3 group C member 1
OAS2, 2'-5'-oligoadenylate synthetase 2
OASL, 2'-5'-oligoadenylate synthetase like
OXER1, oxoeicosanoid receptor 1
PDGFA, platelet derived growth factor subunit A
PLA2G4A, phospholipase A2 group IVA

PLCB1, phospholipase C beta 1
PPP1R12B, protein phosphatase 1 regulatory subunit 12B
PRKCA, protein kinase C alpha
PRKCB, protein kinase C beta
PTGER2, prostaglandin E receptor 2
PTGER4, prostaglandin E receptor 4
PTGIR , prostaglandin I2 receptor
PTGS1 , prostaglandin-endoperoxide synthase 1
PTGS2 , prostaglandin-endoperoxide synthase 2
PTK2, protein tyrosine kinase 2
RAC1, Rac family small GTPase 1
RAF1, Raf-1 proto-oncogene, serine/threonine kinase
RAPGEF2, Rap guanine nucleotide exchange factor 2
RELA, RELA proto-oncogene, NF-kB subunit
RELB, RELB proto-oncogene, NF-kB subunit
RHOA, ras homolog family member A
RIPK1, receptor interacting serine/threonine kinase 1
RIPK2, receptor interacting serine/threonine kinase 2
ROCK2, Rho associated coiled-coil containing protein kinase 2
RPS6KA5, ribosomal protein S6 kinase A5
SHC1, SHC adaptor protein 1
SMAD7, SMAD family member 7
STAT1, signal transducer and activator of transcription 1
STAT2, signal transducer and activator of transcription 2
STAT3, signal transducer and activator of transcription 3
TCF4, transcription factor 4
TGFB1, transforming growth factor beta 1
TGFB2, transforming growth factor beta 2
TGFBR1, transforming growth factor beta receptor 1
TLR1, toll like receptor 1
TLR2, toll like receptor 2
TLR4, toll like receptor 4
TLR5, toll like receptor 5
TLR6, toll like receptor 6
TLR7, toll like receptor 7
TLR8, toll like receptor 8
TNF, tumor necrosis factor
TNFAIP3, TNF alpha induced protein 3
TNFSF14, TNF superfamily member 14
TOLLIP, toll interacting protein
TRADD, TNFRSF1A associated via death domain
TRAF2, TNF receptor associated factor 2
TREM2, triggering receptor expressed on myeloid cells 2
TWIST2, twist family bHLH transcription factor 2
TYROBP, transmembrane immune signaling adaptor TYROBP

S2. Supplemental results

S2.1. Cell viability
Analysis revealed no main effect of either *M. vaccae* NCTC 11659 ($F_{(4, 30)} = 0.8854$, $p = 0.4845$) or LPS ($F_{(1, 30)} = 0.4101$, $p = 0.5268$), nor was there an interaction effect ($F_{(4, 30)} = 0.2806$, $p = 0.8882$; Fig. S1).

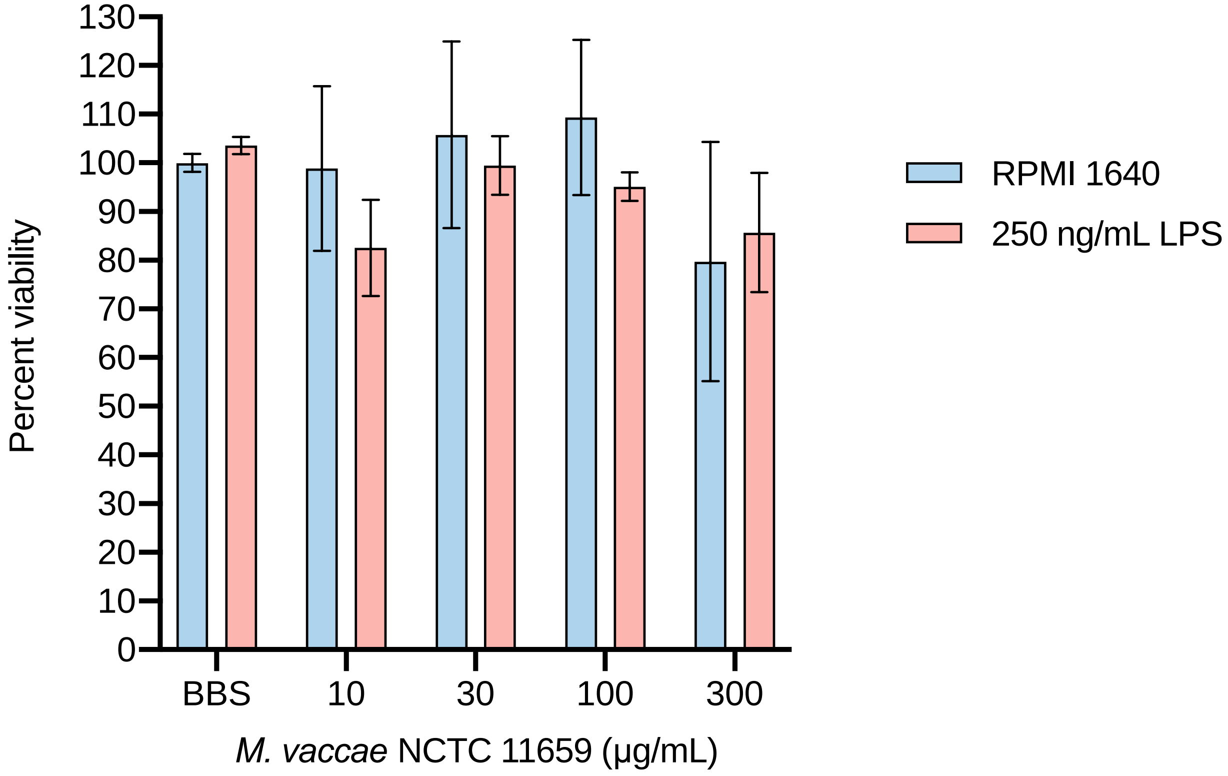

Figure S1. Treating THP-1-derived macrophages with *M. vaccae* NCTC 11659 (0, 10, 30, 100, or 300 µg/mL; 48 h) and lipopolysaccharide (0, 250 ng/mL; 24 h) did not change cell viability. Data are expressed as percent viable cells relative to the BBS/RPMI 1640 condition. Cell viability was assessed by flow cytometry gating of live singlets based on forward and side scatter (FSC/SSC). Sample sizes can be found in Table S1. Abbreviations: BBS, borate-buffered saline; LPS, lipopolysaccharide.

S2.2. Analysis of NanoString housekeeping genes

S2.2.1. CTLC
Analysis revealed an *M. vaccae* NCTC 11659 x LPS interaction ($F_{(2, 29)} = 5.464$, $p = 0.0097$). Dunnett’s multiple comparisons test revealed significant differences between BBS/RPMI 1640 vs. 300 µg/mL *M. vaccae* NCTC 11659/RPMI 1640 ($p = 0.0018$), BBS/RPMI 1640 vs. BBS/250 ng/mL LPS ($p < 0.0001$), 30 µg/mL *M. vaccae* NCTC 11659/RPMI 1640 vs. 30 µg/mL *M. vaccae* NCTC 11659/250 ng/mL LPS ($p < 0.0001$), and 300 µg/mL *M. vaccae* NCTC

11659/RPMI 1640 vs. 300 µg/mL *M. vaccae* NCTC 11659/250 ng/mL LPS ($p = 0.0042$). Fig. S2.

S2.2.2. GAPDH
Analysis revealed an *M. vaccae* NCTC 11659 x LPS interaction ($F_{(2, 29)} = 21.84$, $p < 0.0001$). Dunnett's multiple comparisons test revealed significant differences between BBS/RPMI 1640 vs. BBS/250 ng/mL LPS ($p < 0.0001$), BBS/250 ng/mL LPS vs. 30 µg/mL *M. vaccae* NCTC 11659 ($p < 0.0001$), and BBS/250 ng/mL LPS vs. 300 µg/mL *M. vaccae* NCTC 11659 ($p < 0.0001$; Fig. S2).

S2.2.3. GUSB
Analysis revealed an *M. vaccae* NCTC 11659 main effect ($F_{(2, 29)} = 15.31$, $p < 0.0001$), but no main effect of LPS nor an interaction effect. Dunnett's multiple comparisons test revealed significant differences between BBS/RPMI 1640 vs. 300 µg/mL *M. vaccae* NCTC 11659/RPMI 1640 ($p = 0.0111$) and BBS/250 ng/mL LPS vs. 300 µg/mL *M. vaccae* NCTC 11659/250 ng/mL LPS ($p = 0035$; Fig. S2).

S2.2.4. HPRT1
Analysis revealed an LPS main effect ($F_{(1, 29)} = 36.53$, $p < 0.0001$), but no main effect of *M. vaccae* NCTC 11659 nor an interaction effect. Dunnett's multiple comparisons test revealed significant differences between 30 µg/mL *M. vaccae* NCTC 11659/RPMI 1640 vs. 30 µg/mL *M. vaccae* NCTC 11659/250 ng/mL LPS ($p = 0.0138$) and 300 µg/mL *M. vaccae* NCTC 11659/RPMI 1640 vs. 300 µg/mL *M. vaccae* NCTC 11659/250 ng/mL LPS ($p = 0.0010$; Fig. S2).

S2.2.5. PGK1
Analysis revealed an *M. vaccae* NCTC 11659 main effect ($F_{(2, 29)} = 38.89$, $p < 0.0001$), but no main effect of LPS nor an interaction effect. Dunnett's multiple comparisons test revealed significant differences between BBS/RPMI 1640 vs. 30 µg/mL *M. vaccae* NCTC 11659/RPMI 1640 ($p < 0.0001$), BBS/RPMI 1640 vs. 300 µg/mL *M. vaccae* NCTC 11659/RPMI 1640 ($p < 0.0001$), BBS/250 ng/mL LPS vs. 30 µg/mL *M. vaccae* NCTC 11659/250 ng/mL LPS ($p = 0.0027$), and BBS/250 ng/mL LPS vs. 300 µg/mL *M. vaccae* NCTC 11659/250 ng/mL LPS ($p = 0.0002$; Fig. S2).

S2.2.6. TUBB
Analysis revealed an *M. vaccae* NCTC 11659 x LPS interaction ($F_{(2, 29)} = 4.294$, $p < 0.0233$). Dunnett's multiple comparisons test revealed significant differences between BBS/RPMI 1640 vs. BBS/250 ng/mL LPS ($p < 0.0001$), 30 µg/mL *M. vaccae* NCTC 11659/RPMI 1640 vs. 30 µg/mL *M. vaccae* NCTC 11659/250 ng/mL LPS ($p < 0.0001$), and 300 µg/mL *M. vaccae* NCTC 11659/RPMI 1640 vs. 300 µg/mL *M. vaccae* NCTC 11659/250 ng/mL LPS ($p < 0.0001$; Fig. S2).

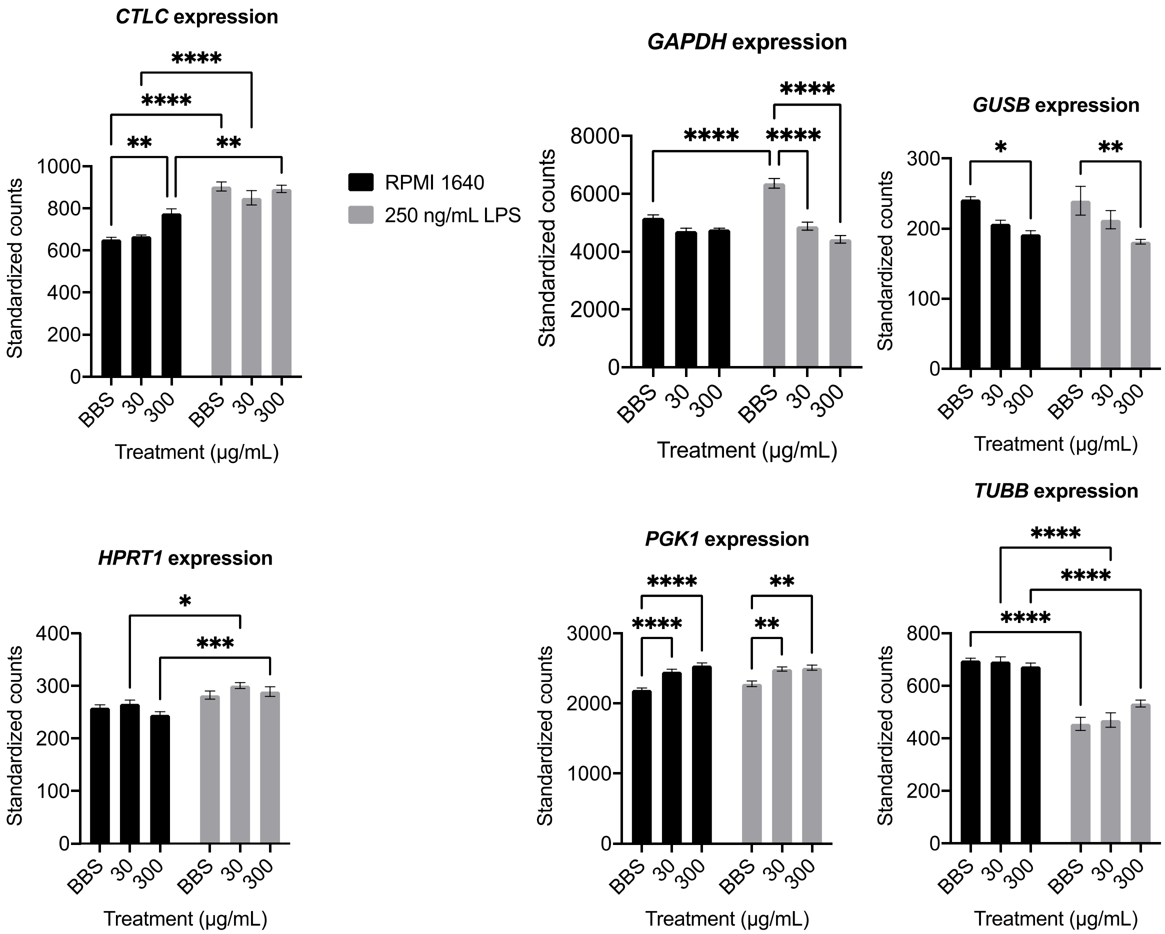

Figure S2. Treating human THP-1 monocyte-derived macrophages with *M. vaccae* NCTC 11659 (48 h) and lipopolysaccharide (LPS; 250 ng/mL, 24 h) resulted in differential expression of all internal housekeeping genes in *Experiment 1*. Abbreviations: BBS, borate-buffered saline; LPS, lipopolysaccharide.

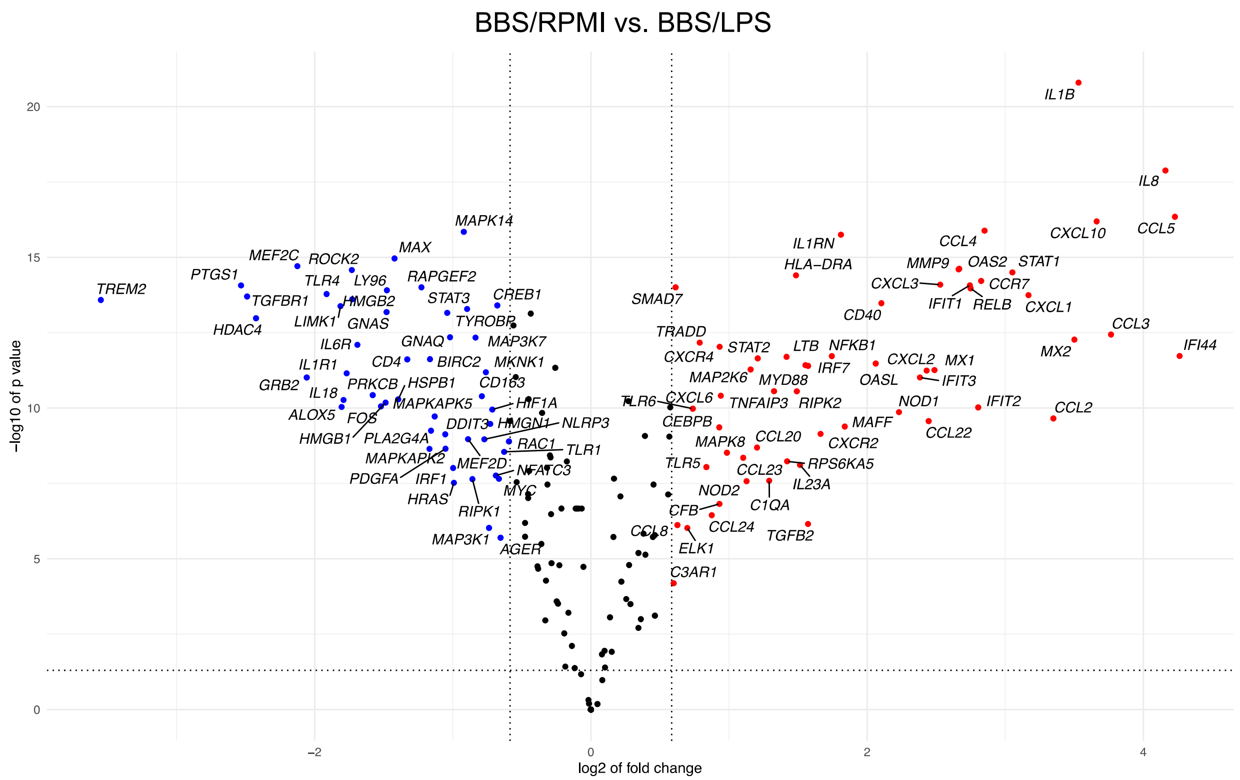

Figure S3. Volcano plot illustrating that lipopolysaccharide (LPS; 250 ng/mL, 24 h) induced an inflammatory gene expression phenotype in human THP-1 derived macrophages. The x axis is the log base 2 of the fold change and the y axis is the negative log base 10 of the *p*-value. Each dot in the figure represents a specific gene. The blue dots in the upper left quadrant of the volcano plot represent the genes that were expressed at lower levels in the BBS vehicle/250 ng/mL LPS group relative to the BBS vehicle/RPMI 1640 vehicle group. The red dots in the upper right quadrant of the volcano plot represent the genes in the BBS vehicle/250 ng/mL LPS group that were expressed at higher levels relative to the BBS vehicle/RPMI 1640 vehicle group. The black dots represent genes that were not differentially expressed between the groups (i.e., the absolute value of the log base 2 of fold change < 1.5 or *p*-value > 0.05). The dashed vertical lines represent log base 2 fold changes of approximately –0.6 or 0.6 (i.e., genes with an absolute fold change of at least 1.5). The horizontal dashed line is the negative log base 10 of *p*-value alpha level of 0.05. The volcano plot was generated using the ggplot2 and ggrepel packages. See *S1. List of abbreviations for all gene symbols* for a complete list of definitions of gene symbols (S1, Supplementary Material online).

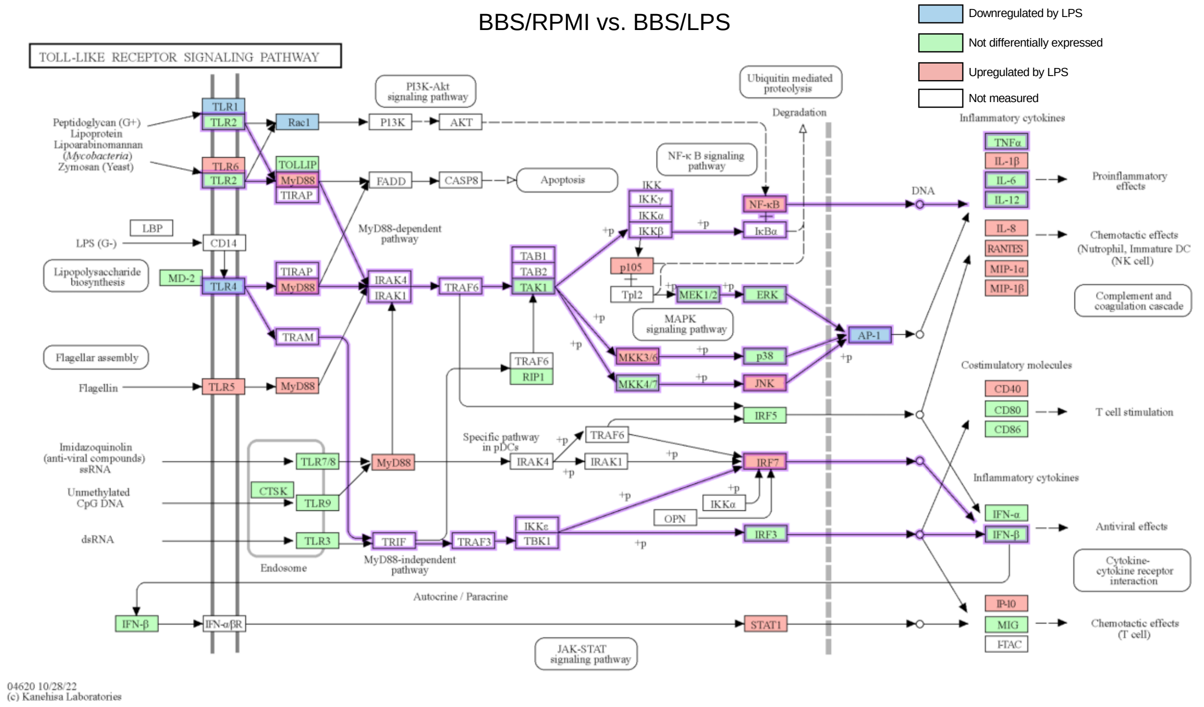

Figure S4. Toll-Like Receptor Signaling KEGG pathway color coded to reflect the effects of 250 ng/mL lipopolysaccharide (LPS). Purple lines indicate TLR2 and TLR4 signaling pathways. Genes highlighted in blue were downregulated by 250 ng/mL LPS, genes highlighted in green were not differentially expressed, genes highlighted in red were upregulated by 250 ng/mL LPS, and genes in white were not measured.

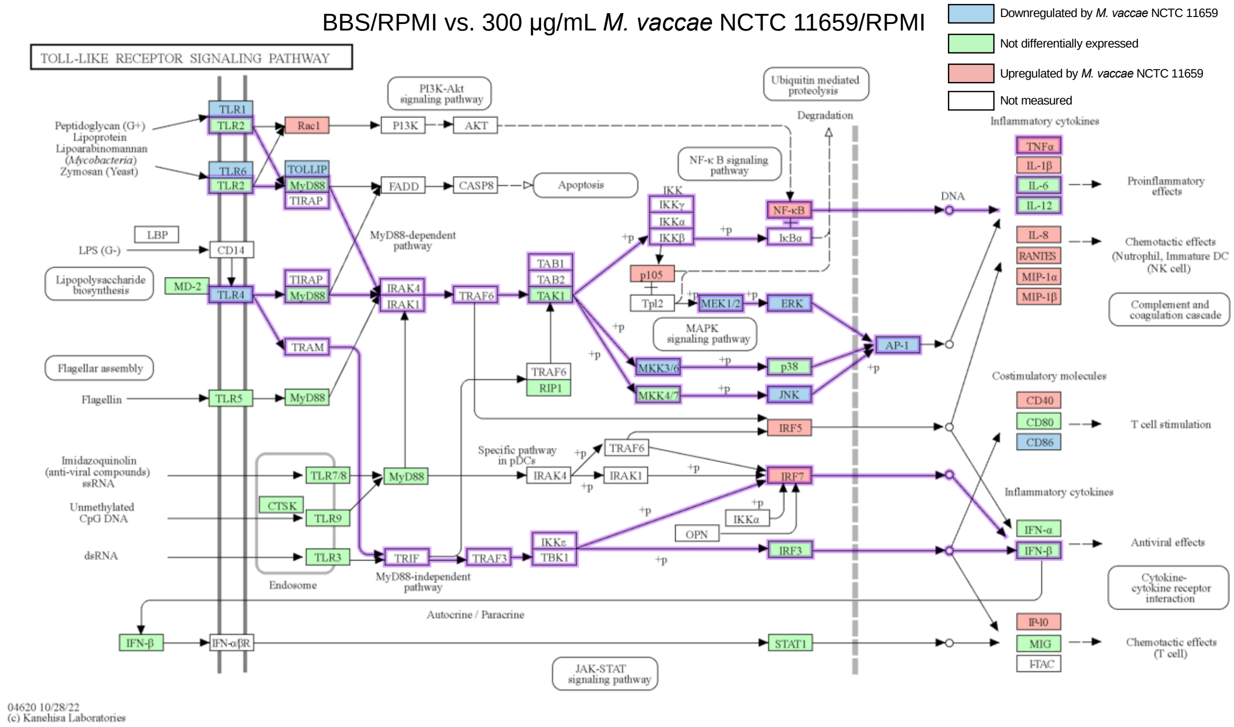

Figure S5. Toll-Like Receptor Signaling KEGG pathway color coded to reflect the effects of 300 µg/mL *M. vaccae* NCTC 11659. Purple lines indicate TLR2 and TLR4 signaling pathways. Genes highlighted in blue were downregulated by 300 µg/mL *M. vaccae* NCTC 11659, genes highlighted in green were not differentially expressed, genes highlighted in red were upregulated by 300 µg/mL *M. vaccae* NCTC 11659, and genes in white were not measured.

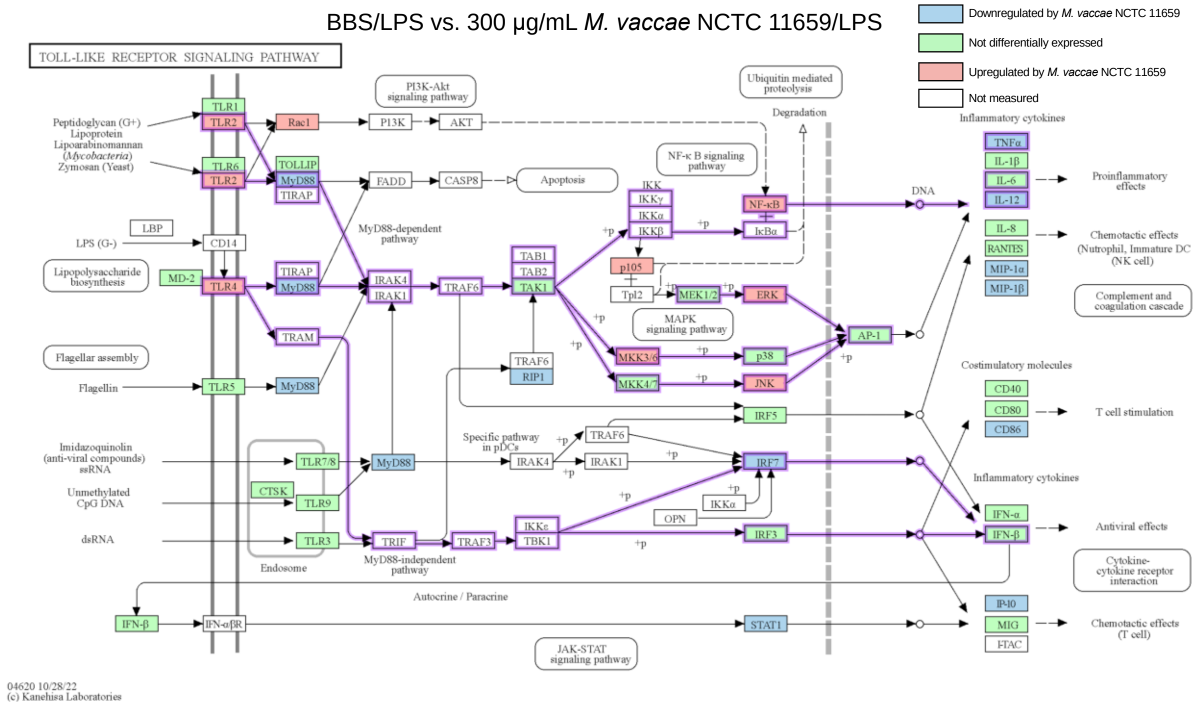

Figure S6. Toll-Like Receptor Signaling KEGG pathway color coded to reflect the effects of *M. vaccae* NCTC 11659 in the presence of 250 ng/mL lipopolysaccharide (LPS). Purple lines indicate TLR2 and TLR4 signaling pathways. Genes highlighted in blue were downregulated by 300 µg/mL *M. vaccae* NCTC 11659, genes highlighted in green were not differentially expressed, genes highlighted in red were upregulated by 300 µg/mL *M. vaccae* NCTC 11659, and genes in white were not measured.

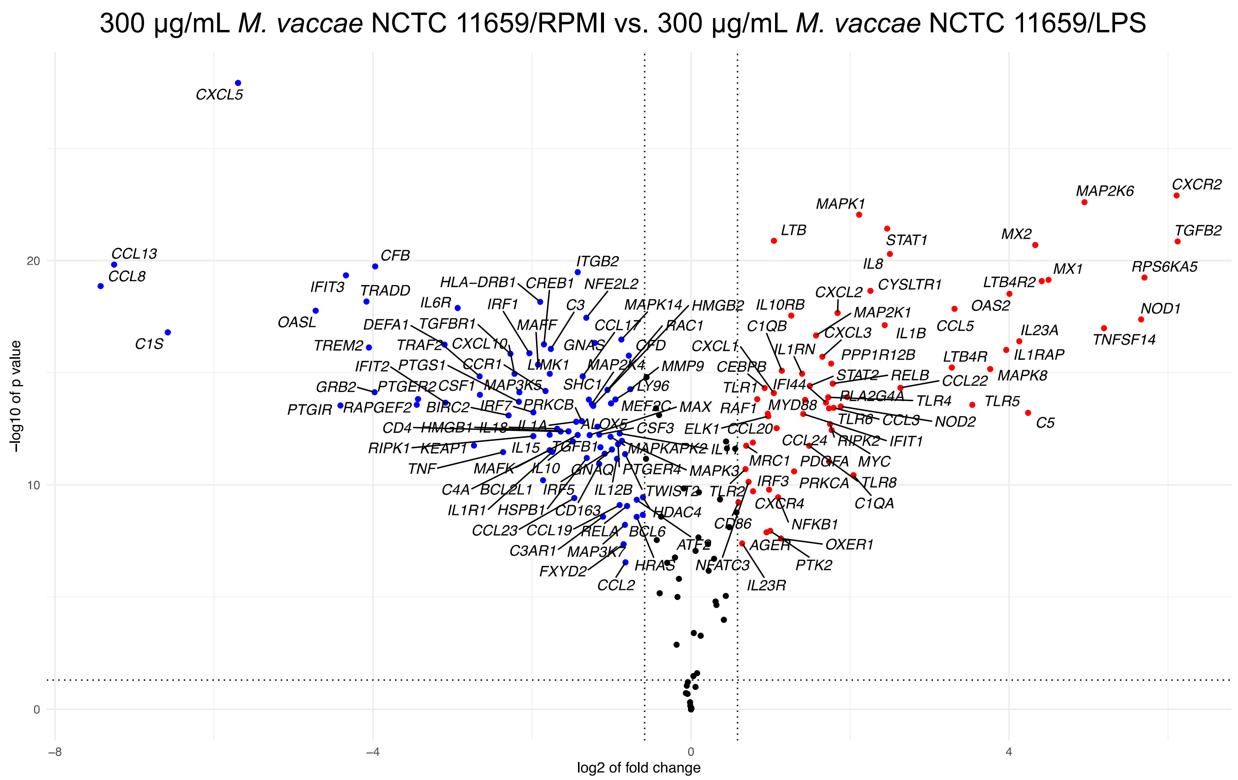

Figure S7. Volcano plot illustrating that lipopolysaccharide (LPS; 250 ng/mL) downregulated a number of NFκB genes among THP-1-derived macrophages previously exposed to *M. vaccae* NCTC 11659. The x axis is the log base 2 of the fold change and the y axis is the negative log base 10 of the *p*-value. Each dot in the figure represents a specific gene. The blue dots in the upper left quadrant of the volcano plot represent the genes that were expressed at lower levels in 300 µg/mL *M. vaccae* NCTC 11659/250 ng/mL LPS group relative to *M. vaccae* NCTC 11659/RPMI 1640 vehicle control group. The red dots in the upper right quadrant of the volcano plot represent the genes in the 300 µg/mL *M. vaccae* NCTC 11659/250 ng/mL LPS group that were expressed at higher levels relative to the 300 µg/mL *M. vaccae* NCTC 11659/RPMI 1640 vehicle control group. The black dots represent genes that were not differentially expressed between the groups (i.e., the absolute value of the log base 2 of fold change < 1.5 or adjusted *p*-value > 0.05). The dashed vertical lines represent log base 2 fold changes of approximately –0.6 or 0.6 (i.e., genes with an absolute fold change of at least 1.5). The horizontal dashed line is the negative log base 10 of *p*-value alpha level of 0.05. The volcano plot was generated using the ggplot2 and ggrepel packages. See *S1. List of abbreviations for all gene symbols* for a complete list of definitions of gene symbols (S1, Supplementary Material online).

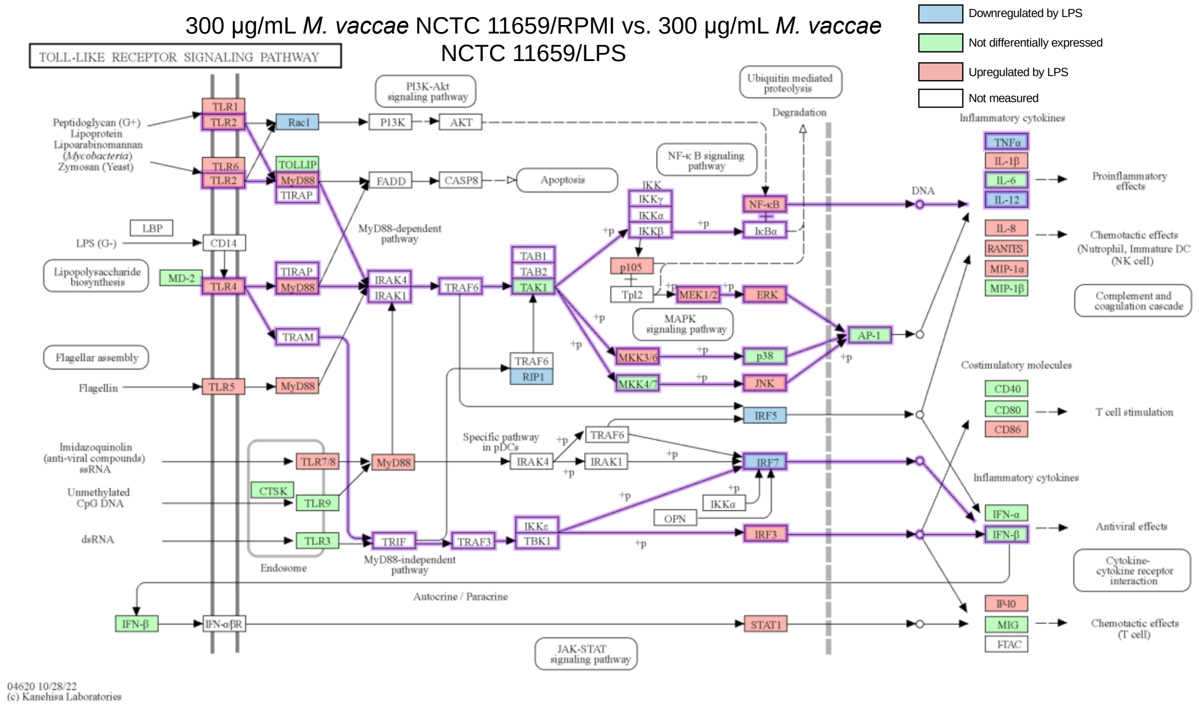

Figure S8. Toll-Like Receptor Signaling KEGG pathway color coded to reflect the effects of 250 ng/mL lipopolysaccharide (LPS) in the presence of 300 µg/mL *M. vaccae* NCTC 11659. Purple lines indicate TLR2 and TLR4 signaling pathways. Genes highlighted in blue were downregulated by 250 ng/mL LPS, genes highlighted in green were not differentially expressed, genes highlighted in red were upregulated by 250 ng/mL LPS, and genes in white were not measured.

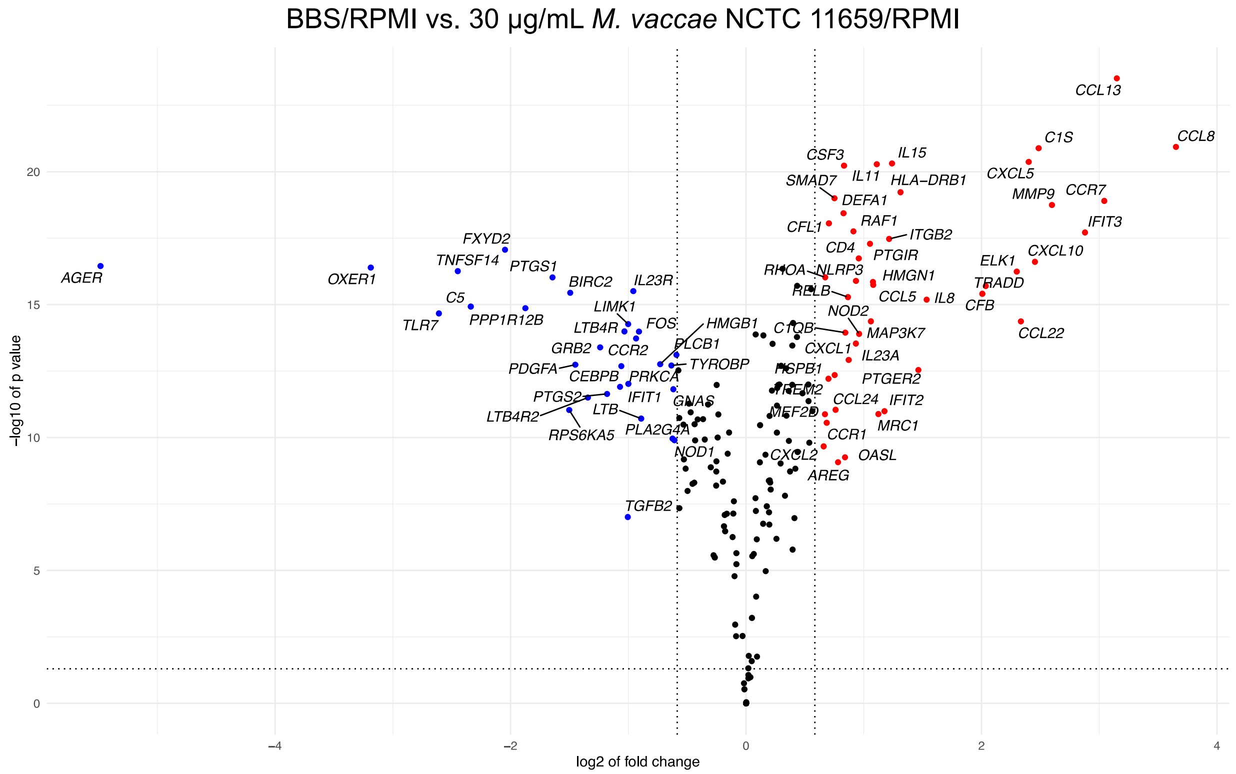

Figure S9. Volcano plot illustrating that, in the RPMI 1640 vehicle control condition, *M. vaccae* NCTC 11659 (30 µg/mL) results in differential expression of both proinflammatory and anti-inflammatory markers in human THP-1-derived macrophages. The x axis is the log base 2 of the fold change and the y axis is the negative log base 10 of the *p*-value. Each dot in the figure represents a specific gene. The blue dots in the upper left quadrant of the volcano plot represent the genes that were expressed at lower levels in 30 µg/mL *M. vaccae* NCTC 11659/RPMI 1640 vehicle group relative to the BBS vehicle/RPMI 1640 vehicle group. The red dots in the upper right quadrant of the volcano plot represent the genes in the 30 µg/mL *M. vaccae* NCTC 11659/RPMI 1640 vehicle group that were expressed at higher levels relative to the BBS vehicle/RPMI 1640 vehicle group. The black dots represent genes that were not differentially expressed between the groups (i.e., the absolute value of the log base 2 of fold change < 1.5 or *p*-value > 0.05). The dashed vertical lines represent log base 2 fold changes of approximately –0.6 or 0.6 (i.e., genes with a fold change of at least 1.5). The horizontal dashed line is the negative log base 10 of adjusted *p*-value alpha level of 0.05. The volcano plot was generated using the ggplot2 and ggrepel packages. See *S1. List of abbreviations for all gene symbols* for a complete list of definitions of gene symbols (S1, Supplementary Material online).

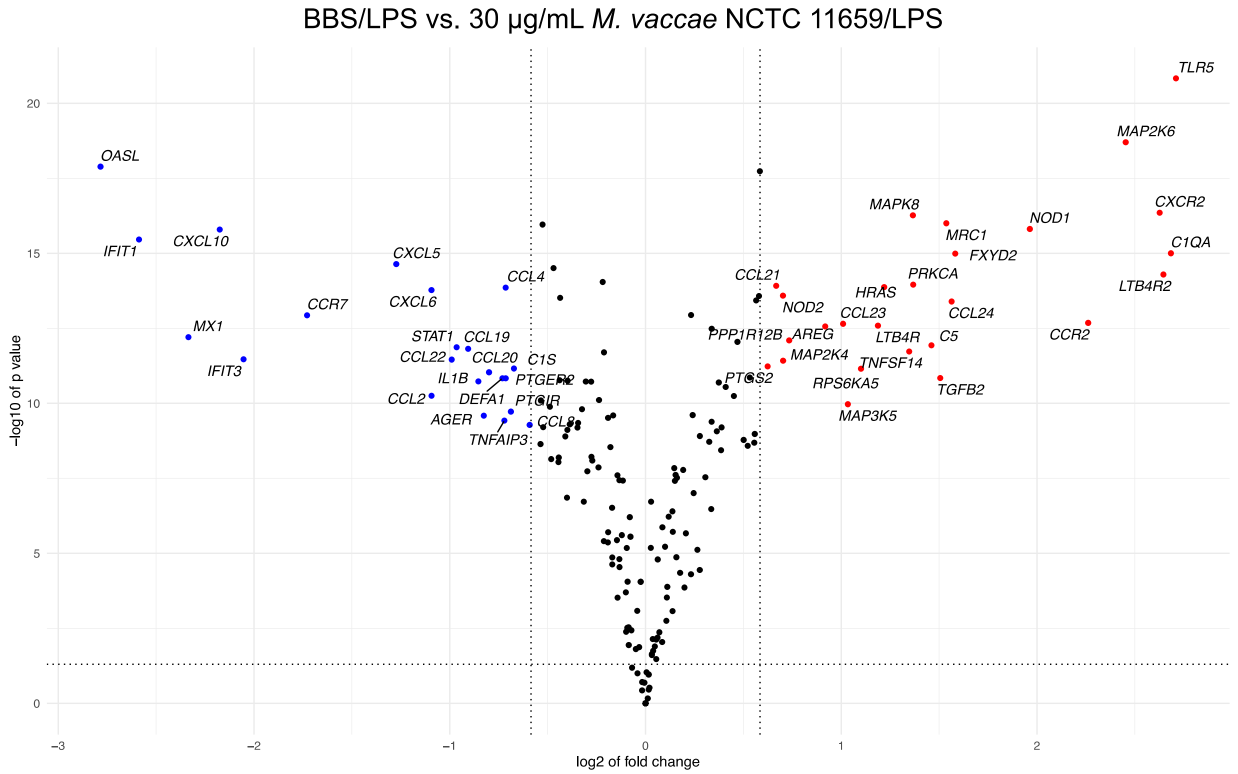

Figure S10. Volcano plot illustrating that, in the lipopolysaccharide (LPS; 250 ng/mL) challenge condition, *M. vaccae* NCTC 11659 (30 µg/mL) shifted human THP-1-derived macrophages toward an anti-inflammatory phenotype, for example, with increased *TGFB2*, *MRC1* expression and decreased *IL1B* and *STAT1* expression when assessed 24 h after LPS challenge. The x axis is the log base 2 of the fold change and the y axis is the negative log base 10 of the *p*-value. Each dot in the figure represents a specific gene. The blue dots in the upper left quadrant of the volcano plot represent the genes that were expressed at lower levels in 30 µg/mL *M. vaccae* NCTC 11659/250 ng/mL LPS group relative to BBS vehicle/250 ng/mL LPS group. The red dots in the upper right quadrant of the volcano plot represent the genes in 30 µg/mL *M. vaccae* NCTC 11659/250 ng/mL LPS group that were expressed at higher levels relative to the BBS vehicle/250 ng/mL LPS group. The black dots represent genes that were not differentially expressed between the groups (i.e., the absolute value of the log base 2 of fold change < 1.5 or adjusted *p*-value > 0.05). The dashed vertical lines represent log base 2 fold changes of approximately –0.6 or 0.6 (i.e., genes with a fold change of at least 1.5). The horizontal dashed line is the negative log base 10 of *p*-value alpha level of 0.05. The volcano plot was generated using the ggplot2 and ggrepel packages. See *S1. List of abbreviations for all gene symbols* for a complete list of definitions of gene symbols (S1, Supplementary Material online).

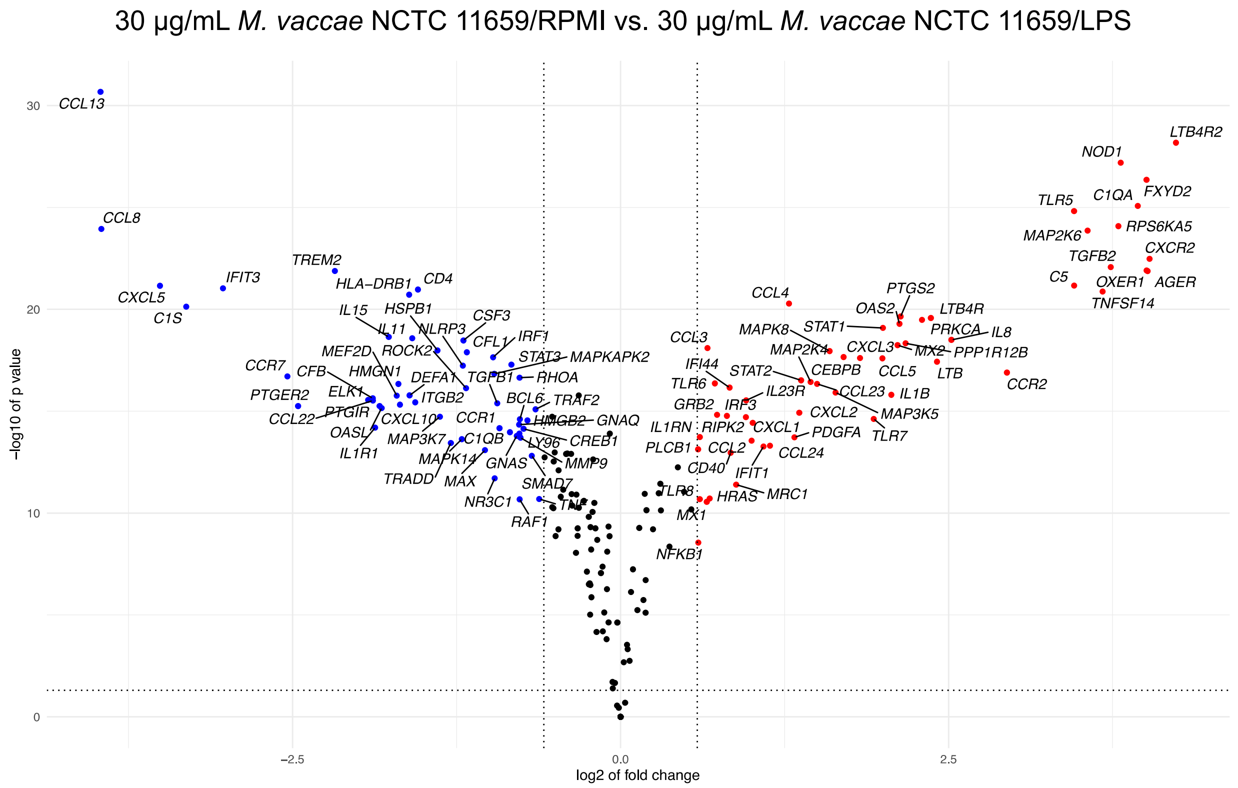

Figure S11. Volcano plot illustrating that, under the *M. vaccae* NCTC 11659 (30 µg/mL) treatment condition, lipopolysaccharide (LPS; 250 ng/mL) downregulated a number of genes involved in the LPS-induced NFκB signaling cascade in THP-1-derived macrophages, including *LY96*, *MAP3K7*, *MAPK14*, *TNF*, and downregulated genes associated with LPS induction of *NLRP3*, a key component of the NLRP3 inflammasome. The x axis is the log base 2 of the fold change and the y axis is the negative log base 10 of the *p*-value. Each dot in the figure represents a specific gene. The blue dots in the upper left quadrant of the volcano plot represent the genes that were expressed at lower levels in 30 µg/mL *M. vaccae* NCTC 11659/250 ng/mL LPS group relative to *M. vaccae* NCTC 11659/RPMI 1640 vehicle control group. The red dots in the upper right quadrant of the volcano plot represent the genes in the 30 µg/mL *M. vaccae* NCTC 11659/250 ng/mL LPS group that were expressed at higher levels relative to the 30 µg/mL *M. vaccae* NCTC 11659/RPMI 1640 vehicle control group. The black dots represent genes that were not differentially expressed between the groups (i.e., the absolute value of the log base 2 of fold change < 1.5 or adjusted *p*-value > 0.05). The dashed vertical lines represent log base 2 fold changes of approximately –0.6 or 0.6 (i.e., genes with a fold change of at least 1.5). The horizontal dashed line is the negative log base 10 of *p*-value alpha level of 0.05. The volcano plot was generated using the ggplot2 and ggrepel packages. See *S1. List of abbreviations for all gene symbols* for a complete list of definitions of gene symbols (S1, Supplementary Material online).

S2.3. Analysis of ACTB housekeeping gene for real time RT-PCR

Analysis revealed a main effect of *M. vaccae* NCTC 11659 ($F_{(4, 134)} = 4.556$, $p = 0.0018$) and LPS ($F_{(3, 134)} = 42.75$, $p < 0.0001$). Dunnett's multiple comparisons test revealed significant differences between BBS/RPMI 1640 and BBS/0.5 ng/mL LPS ($p = 0.0038$), 10 µg/mL *M. vaccae* NCTC 11659/RPMI 1640 and 10 µg/mL *M. vaccae* NCTC 11659/0.5 ng/mL LPS ($p <$

0.0001), 10 µg/mL *M. vaccae* NCTC 11659/RPMI 1640 and 10 µg/mL *M. vaccae* NCTC 11659/2.5 ng/mL LPS ($p < 0.0001$), 10 µg/mL *M. vaccae* NCTC 11659/RPMI 1640 and 10 µg/mL *M. vaccae* NCTC 11659/250 ng/mL LPS ($p = 0.0036$), 30 µg/mL *M. vaccae* NCTC 11659/RPMI 1640 and 30 µg/mL *M. vaccae* NCTC 11659/0.5 ng/mL LPS ($p = 0.0002$), 300 µg/mL *M. vaccae* NCTC 11659/RPMI 1640 and 300 µg/mL *M. vaccae* NCTC 11659/0.5 ng/mL LPS ($p < 0.0001$), and 300 µg/mL *M. vaccae* NCTC 11659/RPMI 1640 and 300 µg/mL *M. vaccae* NCTC 11659/2.5 ng/mL LPS ($p = 0.0095$; Fig. S12).

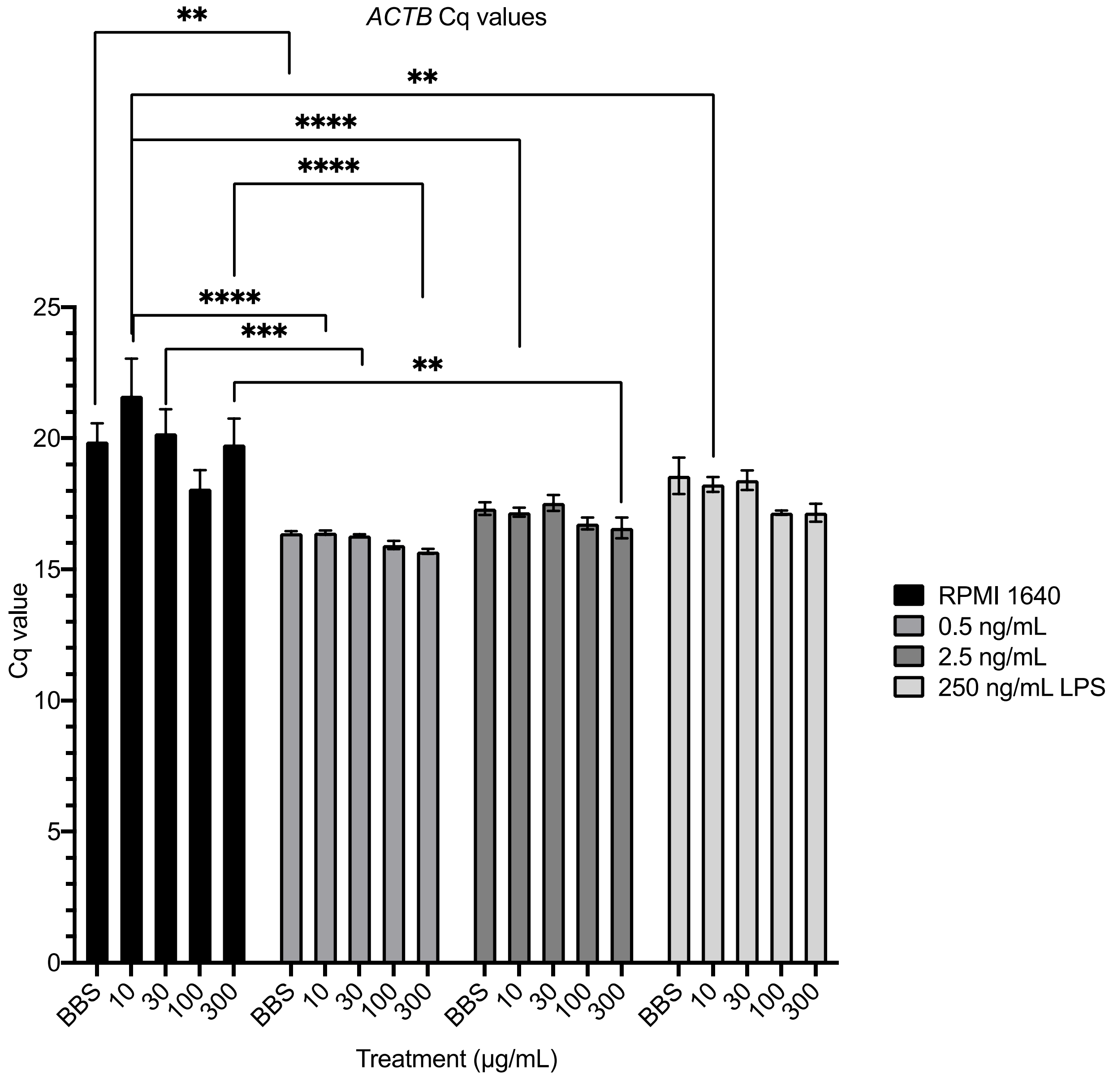

Figure S12. Effects of treatment of THP-1-derived macrophages with *M. vaccae* NCTC 11659 (0, 10, 30, 100, or 300 µg/mL; 48 h) and lipopolysaccharide (0, 250 ng/mL; 24 h) on the expression of *ACTB*. Abbreviations: BBS, borate-buffered saline. LPS, lipopolysaccharide.

S2.4. Validation of effects of heat-killed M. vaccae NCTC 11659 and LPS on proinflammatory IL12A, IL12B, and IL23A gene expression using real time RT-PCR

In the sections below, we describe results from experiments designed to measure expression of genes encoding major inflammatory cytokines in macrophages by extracting total RNA from THP-1 monocyte-derived macrophages and performing real time PCR as described above. Analysis of the housekeeping gene, *ACTB*, for differential expression across treatment groups found no meaningful differences in expression (Fig. S12, Supplementary Material online).

S2.4.1. IL12A mRNA expression

IL-12 production in macrophages is a marker of macrophages with a proinflammatory bias in mice (Orecchioni et al., 2019), and humans (Mantovani et al., 2004). Given IL-12 is a dimer between IL-12p35 (derived from *IL12A*) and IL-12p40 (derived from *IL12B*), *IL12A* and *IL12B* mRNA expression were measured in THP-1 monocyte-derived macrophages after incubating them with various concentrations of *M. vaccae* NCTC 11659 and LPS as described above. Analysis of *IL12A* mRNA expression revealed an *M. vaccae* NCTC 11659 x LPS interaction that approached statistical significance ($F_{(12, 103)} = 1.784$, $p = 0.06$), while main effects of *M. vaccae* NCTC 11659 and LPS were both significant (*M. vaccae* NCTC 11659, $F_{(4, 103)} = 3.732$, $p < 0.01$; LPS, $F_{(3, 103)} = 22.92$, $p < 0.001$). Despite many non-detects in the 0 ng/mL LPS control conditions (i.e., 67.5% of samples were RT-PCR non-detects, Table S1, Supplementary Material online), likely due to the lack of IFN-γ stimulation (Ma et al., 2015;Ma et al., 1996;Grohmann et al., 2001), the trend was for higher concentrations of LPS to increase *IL12A* mRNA expression, and for *M. vaccae* NCTC 11659 to mitigate this effect. Challenging THP-1 monocyte-derived macrophages with 250 ng/mL LPS, relative to the RPMI 1640 control condition, increased *IL12A* mRNA expression in the 300 µg/mL *M. vaccae* NCTC 11659 condition. *M. vaccae* NCTC 11659 had no effect on *IL12A* mRNA expression at any of the concentrations of LPS studied (Fig. S13A, Supplementary Material online). Post hoc *p* values for all planned pairwise comparisons can be found in Table S10, Supplementary Material online.

S2.4.2. IL12B mRNA expression

Analysis of *IL12B* mRNA expression revealed an *M. vaccae* NCTC 11659 x LPS interaction ($F_{(12, 122)} = 13.02$, $p < 0.001$) as well as main effects of *M. vaccae* NCTC 11659 ($F_{(4, 122)} = 6.189$, $p < 0.001$) and LPS ($F_{(3, 122)} = 666.8$, $p < 0.001$). Exposure of THP-1 monocyte-derived macrophages to *M. vaccae* NCTC 11659, in the absence of LPS or at a low concentration of LPS (0.5 ng/mL), increased *IL12B* mRNA expression in a concentration dependent manner (Fig. S13B, Supplementary Material online). Likewise, exposure of THP-1 monocyte-derived macrophages to LPS, in the absence of *M. vaccae* NCTC 11659, increased *IL12B* mRNA expression in a concentration dependent manner (Fig. S13B, Supplementary Material online). However, pretreatment with *M. vaccae* NCTC 11659 attenuated LPS-induced increases in *IL12B* mRNA expression at the highest concentration of LPS studied (i.e., 250 ng/mL; Fig. S13B, Supplementary Material online). Post hoc *p* values for all planned pairwise comparisons can be found in Table S10, Supplementary Material online.

S2.4.3. IL23A mRNA expression

IL-23, a dimer consisting of IL-12p40 and IL-23p19, production in macrophages is a marker of macrophages with a proinflammatory bias (Yamaguchi et al., 2017;Viola et al., 2019), but see (Hou et al., 2018). *IL23A* (encoding IL-23p19) mRNA expression was measured in THP-1 monocyte-derived macrophages after incubating them with various concentrations of *M. vaccae* NCTC 11659 and LPS as described above. Analysis of *IL23A* mRNA expression revealed an *M.*

vaccae NCTC 11659 x LPS interaction ($F_{(12, 140)}$ = 13.88, $p < 0.001$), an *M. vaccae* NCTC 11659 main effect that approached statistical significance ($F_{(4, 140)}$ = 2.083, p = 0.086), and an LPS main effect ($F_{(3, 140)}$ = 13.02, $p < 0.001$). Exposure of THP-1 monocyte-derived macrophages to *M. vaccae* NCTC 11659 in the absence of LPS increased *IL23A* mRNA expression in a concentration dependent manner (Fig. S13C, Supplementary Material online). Likewise, exposure of THP-1 monocyte-derived macrophages to LPS in the absence of *M. vaccae* NCTC 11659 increased *IL23A* mRNA expression in a concentration dependent manner (Fig. S13C, Supplemental Information online). However, pretreatment with *M. vaccae* NCTC 11659 attenuated LPS-induced increases in *IL23A* mRNA expression at the highest concentration of LPS studied (i.e., 250 ng/mL; Fig. S13C, Supplementary Material online). Post hoc *p* values for all planned pairwise comparisons can be found in Table S10, Supplementary Material online.

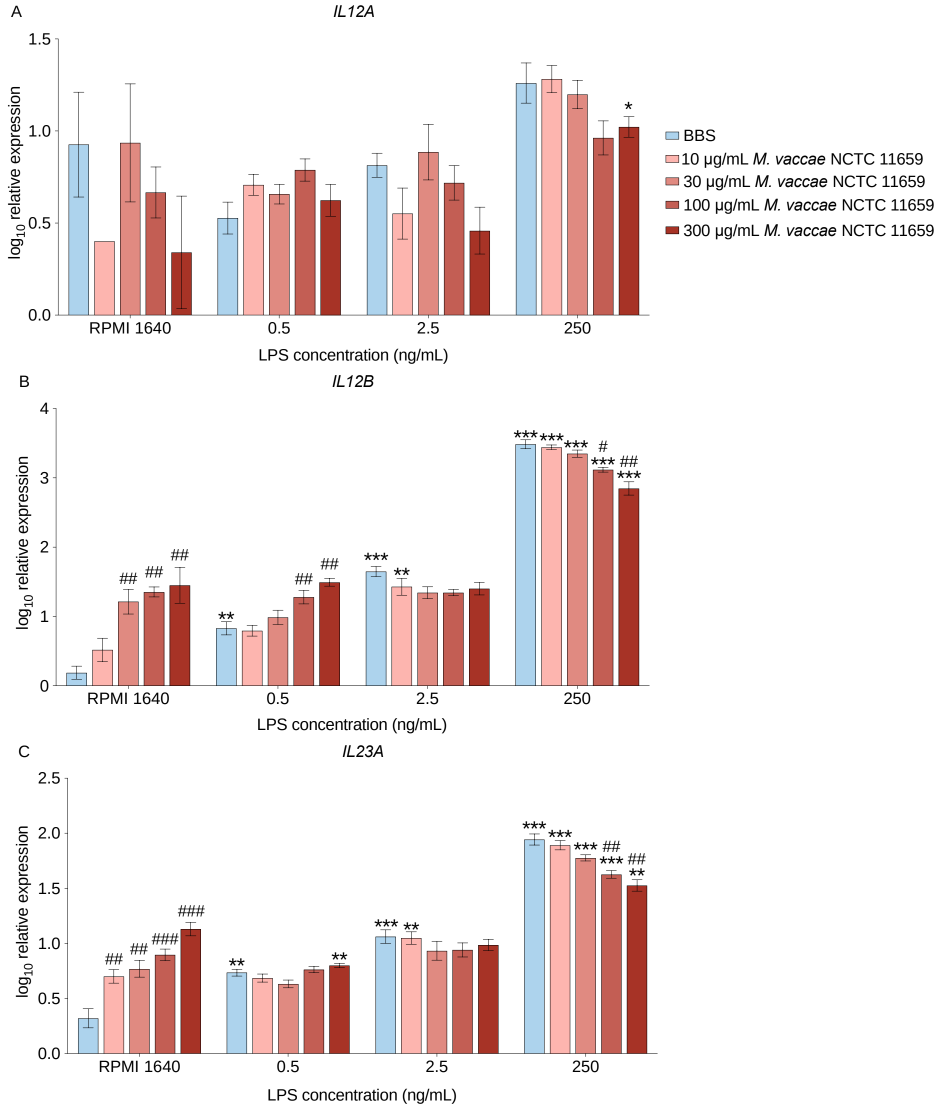

Figure S13. Heat-killed *M. vaccae* NCTC 11659 modulates lipopolysaccharide (LPS)-induced increases in inflammation in THP-1 monocyte-derived macrophages by downregulating inflammatory gene expression. Gene expression was measured using real time reverse

transcription polymerase chain reaction (RT-PCR) and is represented relative to the highest Cq value for each gene using the $2^{-\Delta\Delta Ct}$ method. Data represent means ± SEM. Numbers on the x-axis indicate the concentration of LPS in ng/mL. Data were analyzed using a two-way ANOVA followed by Dunnett's multiple comparisons tests using a single pooled error value from all 20 treatment groups, if appropriate, at a two-tailed alpha level of 0.05. Darker colors represent sequentially higher concentrations of *M. vaccae* NCTC 11659. *$p < 0.05$, **$p < 0.01$, ***$p < 0.001$, effect of LPS, within the same *M. vaccae* NCTC 11659 condition, using RPMI 1640 groups as the comparison groups. #$p < 0.05$, ##$p < 0.01$, ###$p < 0.001$, effect of *M. vaccae* NCTC 11659, within the same LPS condition, using the BBS condition as the comparison group. **A)** *IL12A* mRNA expression. **B)** *IL12B* mRNA expression. **C)** *IL23A* mRNA expression. Abbreviations: BBS, borate-buffered saline; LPS, lipopolysaccharide; NCTC, National Collection of Type Cultures. Sample sizes can be found in Table S1, Supplementary Material online.

S2.5. Effects of heat-killed M. vaccae NCTC 11659 and LPS on IL10 and TGFB1 mRNA expression

IL-10 and TGF-β1 production are biomarkers of macrophages with an anti-inflammatory bias (Viola et al., 2019). *IL10* and *TGFB1* gene expression levels were measured in THP-1 monocyte-derived macrophages after incubating them with various concentrations of *M. vaccae* NCTC 11659 and LPS as described above.

S2.5.1. IL10 mRNA expression

Analysis of *IL10* mRNA expression revealed an *M. vaccae* NCTC 11659 x LPS interaction ($F_{(12, 139)} = 2.971$, $p < 0.001$) as well as main effects of *M. vaccae* NCTC 11659 ($F_{(4, 139)} = 14.81$, $p < 0.001$) and LPS ($F_{(3, 139)} = 4.149$, $p < 0.01$). Exposure of THP-1 monocyte-derived macrophages to *M. vaccae* NCTC 11659 in the absence of LPS increased *IL10* mRNA expression (Fig. S14A, Supplementary Material online). At intermediate concentrations of LPS (i.e., 0.5 and 2.5 ng/mL), *M. vaccae* NCTC 11659 decreased *IL10* mRNA expression (Fig. S14A, Supplementary Material online). Exposure to THP-1-derived macrophages to LPS in the absence of *M. vaccae* NCTC 11659 increased *IL10* mRNA expression in a concentration dependent manner with the exception of the 250 ng/mL concentration of LPS (Fig. S14A, Supplementary Material online). Post hoc *p* values for planned pairwise comparisons can be found in Table S10, Supplemental Information online.

S2.5.2. TGFB1 mRNA expression

Analysis of *TGFB1* mRNA expression revealed an *M. vaccae* NCTC 11659 x LPS interaction ($F_{(12, 137)} = 2.99$, $p < 0.001$) as well as main effects of *M. vaccae* NCTC 11659 ($F_{(4, 137)} = 4.37$, $p < 0.01$) and LPS ($F_{(3, 137)} = 18.86$, $p < 0.001$). Exposure of THP-1 monocyte-derived macrophages to *M. vaccae* NCTC 11659 affected *TGFB1* mRNA expression only in the 2.5 ng/mL LPS condition, where it decreased *TGFB1* mRNA expression in the 30 µg/mL concentration of *M. vaccae* NCTC 11659 and increased *TGFB1* mRNA expression at the highest concentration (300 µg/mL; Fig. S14B, Supplementary Material online). Exposure of THP-1 monocyte-derived macrophages to LPS in the absence of *M. vaccae* NCTC 11659 decreased *TGFB1* expression in the 2.5 ng/mL LPS condition (Fig. S14B, Supplementary Material online). Pretreatment with *M. vaccae* NCTC 11659 (300 µg/mL) attenuated LPS-induced decreases in *TGFB1* mRNA expression in the 2.5 ng/mL LPS condition (Fig. S14B, Supplemental Information online). Post hoc *p* values for planned pairwise comparisons can be found in Table S10, Supplementary Material online.

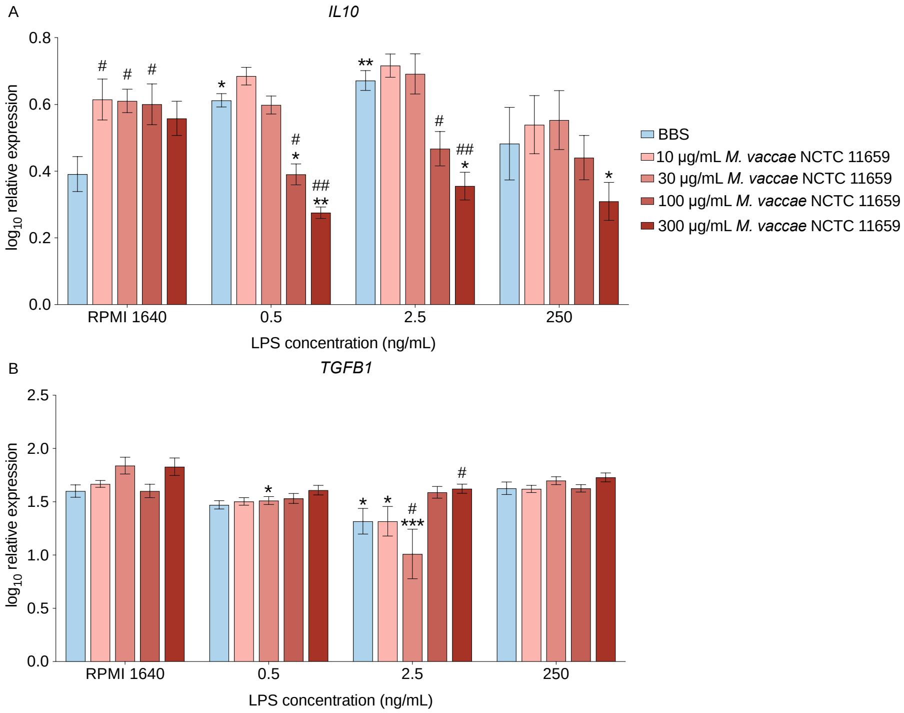

Figure S14. Heat-killed *M. vaccae* NCTC 11659 alone upregulates *IL10* mRNA expression and, among THP-1 monocyte-derived macrophages subsequently stimulated with LPS, downregulates *IL10* mRNA expression and differentially regulates *TGFB1* mRNA expression. Gene expression was measured using real time reverse transcription polymerase chain reaction (RT-PCR) and is represented relative to the highest Cq value for each gene. Data represent means ± SEM. Numbers on the x-axis indicate the concentration of LPS in ng/mL. Data were analyzed using a two-way ANOVA followed by Dunnett's multiple comparisons test using a single pooled error value from all 20 treatment groups, if appropriate, at a two-tailed alpha level of 0.05. Darker colors represent sequentially higher concentrations of *M. vaccae* NCTC 11659. *$p < 0.05$, **$p < 0.01$, ***$p < 0.001$, effect of LPS, within the same *M. vaccae* NCTC 11659 condition, using RPMI 1640 groups as the comparison groups. #$p < 0.05$, ##$p < 0.01$, ###$p < 0.001$, effect of *M. vaccae* NCTC 11659, within the same LPS condition, using the BBS condition as the comparison group. **A)** *IL10* mRNA expression upon challenge with different concentrations of *M. vaccae* NCTC 11659 and LPS. **B)** *TGFB1* mRNA expression upon challenge with different concentrations of *M. vaccae* NCTC 11659 and LPS. Abbreviations: BBS, borate-buffered saline; LPS, lipopolysaccharide; NCTC, National Collection of Type Cultures. Sample sizes can be found in Table S1, Supplemental Information online.

S2.6. Effects of heat-killed M. vaccae NCTC 11659 and LPS on TGFB1:IL12A, TGFB1:IL12B, and TGFB1:IL23A ratios

S2.6.1. TGFB1:IL12A mRNA expression ratio

As mentioned above, expression levels of *IL12A*, *IL12B*, *IL23A*, and *TGFB1* are important in their own right to characterize the immune environment, but the ratio between anti-inflammatory and inflammatory cytokines in the environment is an important feature of macrophage polarization. For example, high expression of IL-12, but low expression of TGF-β1, is characteristic of macrophages with a proinflammatory bias (Mantovani et al., 2004;Viola et al., 2019). In contrast, high expression of TGF-β1, but low expression of IL-12, is characteristic of macrophages with an anti-inflammatory bias, particularly macrophages implicated in anti-inflammatory and immunosuppressive effects (Mantovani et al., 2004;Viola et al., 2019). This suggests that the ratio of *TGFB1* to *IL12A*, *IL12B*, and *IL23A* may be a meaningful measure of macrophage polarization. The ratio of *TGFB1* to *IL12A* expression was calculated in THP-1 monocyte-derived macrophages after incubating them with various doses of *M. vaccae* NCTC 11659 and LPS as described above. In line with low expression of *IL12A* mRNA in the vehicle (0 ng/mL LPS) condition, 67.5% of *TGFB1:IL12A* mRNA expression ratio data were real time RT-PCR non-detects (Table S9, Supplementary Material online). Analysis of the *TGFB1:IL12A* mRNA expression ratio revealed an *M. vaccae* NCTC 11659 x LPS interaction ($F_{(12, 102)} = 2.73$, $p < 0.01$) as well as main effects of *M. vaccae* NCTC 11659 ($F_{(4, 102)} = 6.07$, $p < 0.001$) and LPS ($F_{(3, 102)} = 10.04$, $p < 0.001$). Exposure of THP-1 monocyte-derived macrophages to the highest concentration of *M. vaccae* NCTC 11659 (300 µg/mL) in the presence of 2.5 ng/mL LPS increased the *TGFB1:IL12A* mRNA expression ratio (Fig. S15A). Exposure of THP-1 monocyte-derived macrophages to LPS in the absence of *M. vaccae* NCTC 11659 did not affect the *TGFB1:IL12A* mRNA expression ratio. Post hoc *p* values for planned pairwise comparisons can be found in Table S10, Supplementary Material online.

S2.6.2. TGFB1:IL12B mRNA expression ratio

The ratio of *TGFB1* to *IL12B* expression was calculated in THP-1 monocyte-derived macrophages after incubating them with various doses of *M. vaccae* NCTC 11659 and LPS as described above. Analysis of the *TGFB1:IL12B* mRNA expression ratios revealed an *M. vaccae* NCTC 11659 x LPS interaction ($F_{(12, 119)} = 8.70$, $p < 0.001$); there was no main effect of *M. vaccae* NCTC 11659 ($F_{(4, 119)} = 1.962$, $p = 0.10$), but there was a main effect of LPS ($F_{(3, 119)} = 320.2$, $p < 0.001$). Exposure of THP-1 monocyte-derived macrophages to *M. vaccae* NCTC 11659 in the absence of LPS and in the presence of 0.5 ng/mL LPS dose-dependently decreased the *TGFB1:IL12B* mRNA expression ratio (Fig. S15B). Likewise, exposure of THP-1 monocyte-derived macrophages to LPS in the absence of *M. vaccae* NCTC 11659 dose dependently decreased the *TGFB1:IL12B* mRNA expression ratio (Fig. S15B). In contrast, pretreatment with *M. vaccae* NCTC 11659 increased the *TGFB1:IL12B* mRNA expression ratio in the presence of higher concentrations of LPS (2.5 ng/mL and 250 ng/mL; Fig. S15B), consistent with potential anti-inflammatory effects of *M. vaccae* NCTC 11659 under these conditions. Post hoc *p* values for planned pairwise comparisons can be found in Table S10, Supplementary Material online.

S2.6.3. TGFB1:IL23A mRNA expression ratio

The ratio of *TGFB1* to *IL23A* expression was calculated in THP-1 monocyte-derived macrophages after incubating them with various doses of *M. vaccae* NCTC 11659 and LPS as

described above. Analysis of the *TGFB1:IL23A* mRNA expression ratios revealed an *M. vaccae* NCTC 11659 x LPS interaction ($F_{(12, 137)} = 5.909$, $p < 0.001$)); there was no main effect of *M. vaccae* NCTC 11659 ($F_{(4, 137)} = 1.18$, $p = 0.32$), but there was a main effect of LPS ($F_{(3, 137)} = 123.3$, $p < 0.001$). Exposure of THP-1 monocyte-derived macrophages to *M. vaccae* NCTC 11659 in the absence of LPS dose dependently decreased the *TGFB1:IL23A* mRNA expression ratio (Fig. S15C). Likewise, exposure of THP-1 monocyte-derived macrophages to LPS in the absence of *M. vaccae* NCTC 11659 dose dependently decreased the *TGFB1:IL23A* mRNA expression ratio (Fig. S15C). In contrast, pretreatment with *M. vaccae* NCTC 11659 increased the *TGFB1:IL23A* mRNA expression ratio in the presence of higher concentrations of LPS (2.5 ng/mL and 250 ng/mL; Fig. S15C), consistent with potential anti-inflammatory effects of *M. vaccae* NCTC 11659 under these conditions. Post hoc *p* values for planned pairwise comparisons can be found in Table S10, Supplemental Information online.

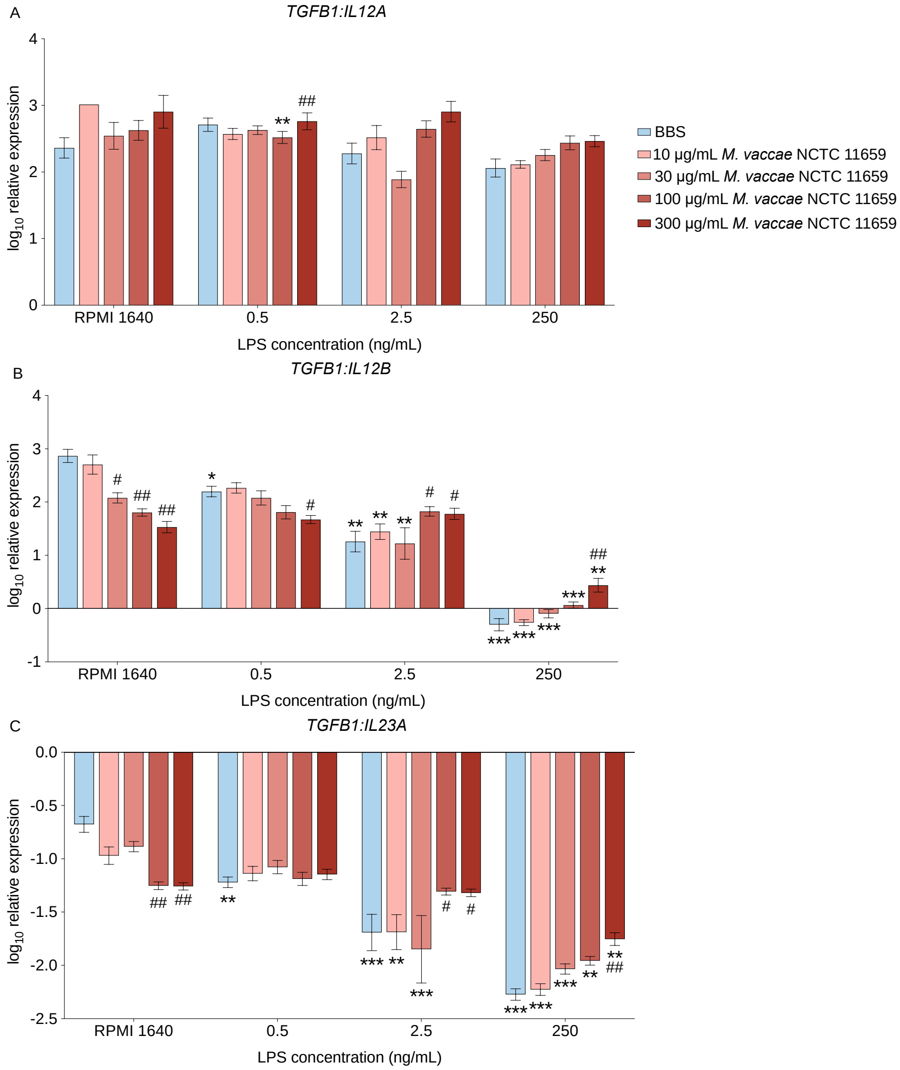

Figure S15. Heat-killed *M. vaccae* NCTC 11659 modulates lipopolysaccharide (LPS)-induced exaggeration of inflammation in THP-1 monocyte-derived macrophages by increasing the ratio of *TGFB1* expression to inflammatory gene expression. Gene expression was measured using real time reverse transcription polymerase chain reaction (RT-PCR) and is represented relative to the highest Cq value for each gene. Data represent means ± SEM. Numbers on the x-axis indicate the concentration of LPS in ng/mL. Data were analyzed using a two-way ANOVA followed by Dunnett's multiple comparisons test using a single pooled error value from all 20 treatment groups, if appropriate, at a two-tailed alpha level of 0.05. Darker colors represent sequentially higher concentrations of *M. vaccae* NCTC 11659. *$p < 0.05$, **$p < 0.01$, ***$p < 0.001$, effect of LPS, within the same *M. vaccae* NCTC 11659 condition, using the RPMI 1640 groups as the comparison groups. #$p < 0.05$, ##$p < 0.01$, ###$p < 0.001$, effect of *M. vaccae* NCTC 11659, within the same LPS condition, using the BBS condition as the comparison group. **A)** *TGFB1:IL12A* mRNA expression ratio upon challenge with different concentrations of *M. vaccae* NCTC 11659 and LPS. **B)** *TGFB1:IL12B* mRNA expression ratio upon challenge with different concentrations of *M. vaccae* NCTC 11659 and LPS. **C)** *TGFB1:IL23A* mRNA expression ratio upon challenge with different concentrations of *M. vaccae* NCTC 11659 and

LPS. Abbreviations: BBS, borate-buffered saline; LPS, lipopolysaccharide; NCTC, National Collection of Type Cultures. Sample sizes can be found in Table S1, Supplemental Information online.

S3. Supplemental Materials and Methods

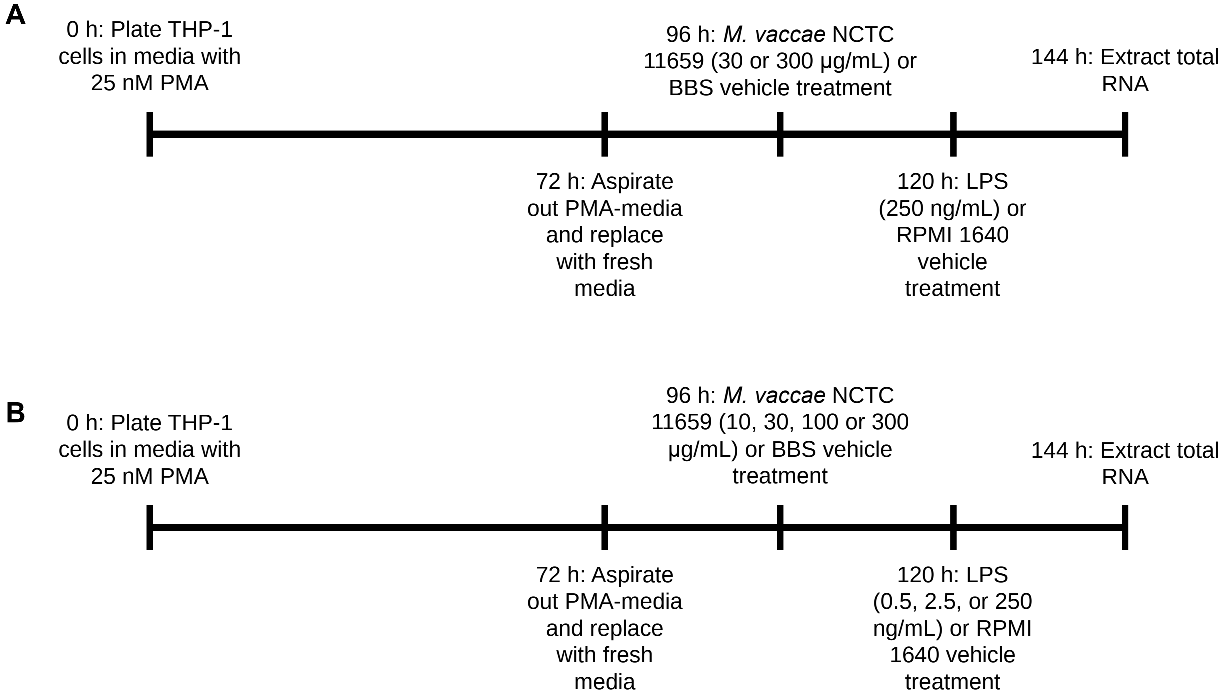

Figure S16. Summary of experimental timelines for (A) *Experiment 1* and (B) *Experiment 2*. Abbreviations: BBS, borate-buffered saline; LPS, lipopolysaccharide; NCTC, National Collection of Type Cultures; PMA, phorbol 12-myristate 13-acetate; RNA, ribonucleic acid.

S3.1. Flow cytometry for assessment of cell viability

Cell plates containing 525 µL/well were centrifuged at 500 x g for 5 minutes at room temperature. After removing cell media, adherent differentiated cells were collected by pipetting 0.5 mL of 5 mM ethylenediaminetetraacetic acid-phosphate buffered solution (EDTA, Cat. No. E4884, Sigma-Aldrich; PBS: sodium chloride, Cat. No. BP358-212, Fisher Scientific, Fair Lawn, New Jersey, USA; potassium phosphate monobasic, Cat. No. P286-1, Fisher Scientific; sodium phosphate dibasic anhydrous, Cat. No. S374-500, Fisher Scientific; potassium chloride, Cat. No. P217-500, Fisher Scientific) into each well and pipetting up and down. Cell viability was assessed by flow cytometry gating of live singlets based on forward and side scatter (FSC/SSC). Flow cytometry was performed with an independent set of cells separate from those used for real time RT-qPCR. Sample sizes for flow cytometry studies are listed in Table S1. Flow cytometry was conducted using a BD Special Order FACSArrayTM Bioanalyzer machine (serial no. W91100224, BD Biosciences, Franklin Lakes, NJ, USA). All downstream analysis was carried out using the FlowJo Software (version 10.1, FlowJo, LLC., Ashland, OR, USA).

S3.2. Flow cytometry statistical analysis

Flow cytometry data for cell viability analysis were represented as the percent of cells viable to the mean of the BBS vehicle/RPMI 1640 vehicle group, which was done by taking the gate for live cells. Flow cytometry data were analyzed using two-way ANOVA followed by Dunnett's multiple comparisons test, if appropriate, at a two-tailed alpha level of 0.05.

Table S1. Sample sizes (*n*) for the flow cytometry experiment cell viability in response to *M. vaccae* NCTC 11659 and lipopolysaccharide (LPS). Sample size represents 1 experimental replicate.

**LPS Treatment**	**Bacterial Treatment**	**Sample Size (*n*)**
0 ng/mL	BBS	5
	10 µg/mL *M. vaccae* NCTC 11659	5
	30 µg/mL *M. vaccae* NCTC 11659	5
	100 µg/mL *M. vaccae* NCTC 11659	5
	300 µg/mL *M. vaccae* NCTC 11659	5
250 ng/mL	BBS	4
	10 µg/mL *M. vaccae* NCTC 11659	4
	30 µg/mL *M. vaccae* NCTC 11659	4
	100 µg/mL *M. vaccae* NCTC 11659	4
	300 µg/mL *M. vaccae* NCTC 11659	4
0 ng/mL	BBS	5
	10 µg/mL *M. vaccae* NCTC 11659	5
	30 µg/mL *M. vaccae* NCTC 11659	5
	100 µg/mL *M. vaccae* NCTC 11659	5
	300 µg/mL *M. vaccae* NCTC 11659	5
250 ng/mL	BBS	4
	10 µg/mL *M. vaccae* NCTC 11659	4
	30 µg/mL *M. vaccae* NCTC 11659	4
	100 µg/mL *M. vaccae* NCTC 11659	4
	300 µg/mL *M. vaccae* NCTC 11659	4

Table S2. Fold changes among differentially expressed genes for the BBS/250 ng/mL LPS vs BBS/RPMI 1640 comparison in *Experiment 1* using the NanoString platform.

Gene symbol	log2FoldChange
Upregulated	
IFI44	4.262269026
CCL5	4.228568845
IL8	4.159731409
CCL3	3.765570054
CXCL10	3.661646101
IL1B	3.530651461
MX2	3.500801064
CCL2	3.348462814
CXCL1	3.16835941
STAT1	3.051809072
CCL4	2.850338226
CCR7	2.825718296
IFIT2	2.804308939
RELB	2.748962417
IFIT1	2.743794411
OAS2	2.667257557
MMP9	2.662598429
CXCL3	2.529580501
MX1	2.487862151
CCL22	2.445586712
CXCL2	2.431240533
IFIT3	2.382447108
NOD1	2.230599396
CD40	2.103029474
OASL	2.062527622
MAFF	1.837931872
IL1RN	1.810310897
NFKB1	1.744320545
CXCR2	1.663204849
IRF7	1.572842636
TGFB2	1.572199863
MYD88	1.55351638
IL23A	1.514079188
RIPK2	1.491068592
HLA-DRA	1.485166012

RPS6KA5	1.420754255
LTB	1.416792999
TNFAIP3	1.326074938
C1QA	1.291248673
STAT2	1.207951243
CCL20	1.203041045
MAP2K6	1.156471185
NOD2	1.126930781
CCL23	1.102917155
MAPK8	0.985073941
CXCL6	0.940690292
CXCR4	0.932173675
CFB	0.930713986
CEBPB	0.92956128
CCL24	0.875357784
TLR5	0.83638696
TRADD	0.787948598
TLR6	0.737944965
ELK1	0.697904269
CCL8	0.626491937
SMAD7	0.613347757
C3AR1	0.599176377
Downregulated	
TREM2	-3.548187492
PTGS1	-2.532871722
TGFBR1	-2.489080777
HDAC4	-2.424431522
MEF2C	-2.125305204
GRB2	-2.057306367
TLR4	-1.913291151
LIMK1	-1.812929182
ALOX5	-1.804872332
IL18	-1.790840137
IL1R1	-1.76824
ROCK2	-1.730590858
HMGB2	-1.727870056
IL6R	-1.690664298
PRKCB	-1.579542716
FOS	-1.520073498

HMGB1	-1.48640968
GNAS	-1.479297821
LY96	-1.476827195
MAX	-1.421829881
HSPB1	-1.394916551
CD4	-1.329394552
RAPGEF2	-1.226831758
MAPKAPK2	-1.168885773
BIRC2	-1.164865848
PLA2G4A	-1.157716781
MAPKAPK5	-1.130769454
DDIT3	-1.054315672
PDGFA	-1.052492146
TYROBP	-1.040038705
GNAQ	-1.019696587
IRF1	-0.997193312
HRAS	-0.991733602
MAPK14	-0.920002433
STAT3	-0.895976194
MEF2D	-0.888962215
RIPK1	-0.85750619
MAP3K7	-0.835212496
CD163	-0.789549572
NLRP3	-0.77122709
MKNK1	-0.760232478
MAP3K1	-0.737623351
HMGN1	-0.728439309
HIF1A	-0.713836198
NFATC3	-0.687810068
CREB1	-0.677395388
MYC	-0.665728171
AGER	-0.653885323
TLR1	-0.627990698
RAC1	-0.593136501

Table S3. Fold changes among differentially expressed genes for the 300 µg/mL *M. vaccae* NCTC 11659/RPMI 1640 vs BBS/RPMI 1640 comparison in *Experiment 1* using the NanoString platform.

Gene symbol	log2FoldChange
Upregulated	
CCL8	4.89690652
CFB	4.430250149
CCL13	3.870692489
MAFF	3.860842614
IFIT2	3.720039294
MMP9	3.520145054
IFIT3	3.431284527
KEAP1	3.360990863
IL10	3.239398915
C1S	2.862037841
CXCL1	2.782482056
IRF7	2.763613935
CXCL5	2.747719032
CD40	2.639664337
IFI44	2.528043214
TRADD	2.427985433
OASL	2.376190291
IL8	2.225557551
RELB	2.159524239
NFKB1	2.10983004
HLA-DRA	1.910535992
RELA	1.905547488
MAPK3	1.807589712
MAP3K5	1.767905581
CXCL10	1.745745502
C3	1.67081143
SMAD7	1.592342315
CD163	1.547699987
MRC1	1.547476852
ALOX5	1.533939839
RAPGEF2	1.512887703
ITGB2	1.51025086
TRAF2	1.484385294
HLA-DRB1	1.41103933

CXCR4	1.385213822
IL15	1.341519769
IL1B	1.321287767
IL6R	1.307791409
NFE2L2	1.257189249
RHOA	1.224671416
PTGER4	1.218333365
CCL2	1.211098382
GNAQ	1.196668767
IL11	1.196521436
AREG	1.177134361
DAXX	1.176135067
RAC1	1.161219841
CCL3	1.098591596
CCL5	1.055958887
C3AR1	1.033466277
CCL4	1.019245099
TNF	1.010907794
PTGIR	0.993283114
CD4	0.961464272
CSF3	0.889676629
DEFA1	0.884852623
TGFBR1	0.881763235
NFATC3	0.840590723
IRF5	0.801880014
CFD	0.772920032
HSPB1	0.756997065
NOD2	0.745261634
CXCL2	0.743951576
CCR1	0.736963034
ELK1	0.698647747
PRKCB	0.645457851
FXYD2	0.621906369
LTB	0.619950676
Downregulated	
PLA2G4A	-5.144740794
TNFSF14	-5.100462031
MX1	-5.005120687
AGER	-4.778907627

RPS6KA5	-3.392627591
PPP1R12B	-3.33328838
C5	-3.032903984
TGFB2	-2.902286516
IL1RAP	-2.55178396
TLR4	-2.534433312
PTGS2	-2.531511955
OXER1	-2.513443302
NOD1	-2.362597471
PDGFA	-2.359872495
MAP2K1	-2.139549594
TLR5	-1.972131517
OAS2	-1.789295679
CYSLTR1	-1.71961854
IFIT1	-1.697762641
LTB4R	-1.619607434
PRKCA	-1.613126296
FOS	-1.524388349
HRAS	-1.498999247
TCF4	-1.435626898
TLR1	-1.419172567
CXCR2	-1.3854188
MAPK8	-1.287056878
C1QB	-1.28606134
CXCL6	-1.177120774
MYC	-1.174193238
TLR6	-1.125992285
MAX	-1.112550891
CD86	-1.103115614
MEF2C	-1.085875385
IL23R	-1.039724013
TLR7	-0.995868956
MAP2K6	-0.973545549
LTB4R2	-0.962118237
HIF1A	-0.925925934
MAPK1	-0.824273447
MX2	-0.776580478
MKNK1	-0.772712802
IL1R1	-0.767024105

MAPKAPK2	-0.757813202
TOLLIP	-0.755326387
HMGB1	-0.753189183
IL18	-0.737871444
CCR2	-0.733063666
C1QA	-0.723428349
HMGB2	-0.672598711
CCL22	-0.647010509
PTK2	-0.608116411

Table S4. Fold changes among differentially expressed genes for the 300 µg/mL *M. vaccae* NCTC 11659/250 ng/mL LPS vs BBS/250 ng/mL LPS comparison in *Experiment 1* using the NanoString platform.

Gene symbol	log2FoldChange
Upregulated	
LTB4R2	3.087050466
CXCR2	3.05915473
MAP2K6	2.819087782
NFATC3	2.25194415
IL23A	2.247266952
ALOX5	2.16121367
IL1RAP	1.738176198
MRC1	1.681382612
MAPK1	1.648627325
TGFB2	1.64577741
C5	1.593725862
NOD2	1.50209677
MAPK8	1.491295888
NFKB1	1.460314226
TLR8	1.447373153
LTB4R	1.404433165
HDAC4	1.402348842
MAPK3	1.291189699
IL10	1.289208553
MAP3K1	1.270090708
MYC	1.26044476
CXCR4	1.232853584
GNAQ	1.221348027
IL10RB	1.215773582
RELB	1.193184076
CD163	1.181033116
TLR4	1.104777136
TGFBR1	1.099294031
ROCK2	1.088525522
RHOA	1.080929738
NOD1	1.068606356
SMAD7	1.055497516
CYSLTR1	0.986892275
C2	0.986476297

MAPKAPK5	0.976870297
ELK1	0.971771149
KEAP1	0.959985562
RPS6KA5	0.889956977
PRKCB	0.850548766
HSPB1	0.839812465
MAFG	0.832517482
DAXX	0.757933477
MEF2D	0.739331706
TLR5	0.729232422
TLR2	0.723843162
CCL21	0.710800392
RAC1	0.703406301
AREG	0.687881125
CXCL1	0.655032692
CD4	0.650741458
RELA	0.637369019
CFL1	0.617930885
Downregulated	
OASL	-4.408160277
CXCL10	-4.136442755
C1S	-3.506510094
PTGIR	-3.30655998
IFIT3	-3.292122002
AGER	-3.175492742
CXCL5	-3.164124162
CCL8	-3.156239486
IFIT1	-3.030267348
MX1	-2.996931999
CCL2	-2.96239435
PTGER2	-2.942304023
CSF1	-2.900074092
CCL13	-2.842854299
CCL23	-2.785297352
PTGS2	-2.485993629
TRADD	-2.44295147
IFIT2	-2.175390748
DEFA1	-2.162019224
CXCL6	-2.112661844

GRB2	-2.085292422
MAP2K4	-2.033403888
PLA2G4A	-2.022961449
CCR7	-1.975461962
C4A	-1.77929554
TRAF2	-1.742686734
OXER1	-1.65951614
MAFK	-1.49331069
TCF4	-1.452849745
IL1A	-1.445212836
TNF	-1.433492677
CCL4	-1.379946591
BCL2L1	-1.369081025
BIRC2	-1.363869271
CCL17	-1.362721544
RIPK1	-1.289955882
HRAS	-1.191350109
SHC1	-1.089279385
STAT1	-1.084944227
TNFAIP3	-1.047367283
PPP1R12B	-1.033699962
MAX	-0.966200409
IL12B	-0.932718828
CCR1	-0.901525584
TGFB1	-0.887321389
TREM2	-0.883598153
CCL3	-0.873193115
CXCL3	-0.843755424
TWIST2	-0.830382558
HMGB1	-0.808826214
IRF7	-0.793069876
CREB1	-0.765847083
IL1R1	-0.744521002
MAP3K5	-0.735381496
CCL19	-0.726045691
RAPGEF2	-0.710950932
MYD88	-0.696538117
FXYD2	-0.680591194
C3AR1	-0.673336189

IL18	-0.631318416
CD86	-0.608851128
MAPKAPK2	-0.601598776
ATF2	-0.595549675

Table S5. Fold changes among differentially expressed genes for the 300 µg/mL *M. vaccae* NCTC 11659/250 ng/mL LPS vs 300 *M. vaccae* NCTC 11659/RPMI 1640 comparison in *Experiment 1* using the NanoString platform.

Gene symbol	log2FoldChange
Upregulated	
TGFB2	6.120263788
CXCR2	6.107778378
RPS6KA5	5.703338822
NOD1	5.661803223
TNFSF14	5.194677406
MAP2K6	4.949104517
MX1	4.49605084
LTB4R2	4.41108765
MX2	4.329610799
C5	4.239846908
IL23A	4.128700691
OAS2	4.004979806
IL1RAP	3.96485466
MAPK8	3.763426708
TLR5	3.537750899
CCL5	3.313953309
LTB4R	3.280034883
CCL22	2.633775154
IL8	2.501388071
STAT1	2.467503503
IL1B	2.436145023
CYSLTR1	2.25625754
MAPK1	2.114514958
C1QA	2.043388814
PLA2G4A	1.964062564
NOD2	1.883765917
CXCL2	1.843106528
CCL3	1.793785343
RELB	1.782622255
MYC	1.768909827
PPP1R12B	1.762143979
RIPK2	1.747898262
PDGFA	1.737370787
TLR6	1.73506275

TLR4	1.725919297
IFI44	1.697092244
CXCL3	1.651744662
MAP2K1	1.572268911
STAT2	1.493993137
TLR8	1.485784135
MYD88	1.434305325
IFIT1	1.411289703
IL1RN	1.396065573
PRKCA	1.296344054
IL10RB	1.260366684
C1QB	1.1416171
OXER1	1.130713266
NFKB1	1.094804731
CCL24	1.076429621
LTB	1.042537016
CXCL1	1.040910047
PTK2	0.995532526
IRF3	0.981334578
ELK1	0.971027671
RAF1	0.964761299
AGER	0.949529562
CEBPB	0.92649671
TLR1	0.832113446
CXCR4	0.779813437
CCL20	0.778011098
NFATC3	0.723543359
MRC1	0.6933714
TLR2	0.685572337
IL23R	0.643337688
CD86	0.596446442
Downregulated	
CCL8	-7.426654069
CCL13	-7.257435442
C1S	-6.581855389
CXCL5	-5.69820227
OASL	-4.721822945
PTGIR	-4.410253909
IFIT3	-4.340959421

TRADD	-4.082988306
TREM2	-4.052520025
GRB2	-3.981070039
CFB	-3.97321765
RAPGEF2	-3.450670393
PTGER2	-3.434476009
DEFA1	-3.100752779
IFIT2	-3.091121102
IL6R	-2.936137512
KEAP1	-2.731145869
TRAF2	-2.658961131
CSF1	-2.655672587
TNF	-2.364319991
BIRC2	-2.295225331
TGFBR1	-2.27154998
CXCL10	-2.220542155
PTGS1	-2.166346112
MAP3K5	-2.159156417
IRF1	-2.033382401
RIPK1	-1.984652105
IRF7	-1.983841175
MAFF	-1.925089041
HLA-DRB1	-1.897946816
BCL2L1	-1.861726507
CREB1	-1.849988742
CCR1	-1.831054792
C4A	-1.77929554
IL15	-1.778732694
LIMK1	-1.776971678
C3	-1.763476573
IL1R1	-1.745736897
IL18	-1.684287109
CD4	-1.640117366
HMGB1	-1.542046711
IL10	-1.487204801
CCL23	-1.468594605
IL1A	-1.445212836
MAFK	-1.426007454
ITGB2	-1.425378067

PRKCB	-1.374451801
CCL17	-1.362721544
NFE2L2	-1.317708723
HSPB1	-1.312101151
SHC1	-1.285676915
MAX	-1.275479399
MAP2K4	-1.247011546
HMGB2	-1.230519584
GNAS	-1.21140752
ALOX5	-1.177598501
CD163	-1.156216443
CSF3	-1.154071013
TGFB1	-1.13997757
C3AR1	-1.107626089
IRF5	-1.086050172
RAC1	-1.050950041
MAPKAPK2	-1.012671347
MEF2C	-1.006319544
GNAQ	-0.995017327
LY96	-0.951152454
IL12B	-0.932718828
PTGER4	-0.919174883
CCL19	-0.898000527
IL11	-0.896658724
MAPK14	-0.876669289
MAPK3	-0.869578113
FXYD2	-0.848908775
MAP3K7	-0.831733933
TWIST2	-0.830382558
CCL2	-0.825029917
RELA	-0.804637744
CFD	-0.783074509
MMP9	-0.763854742
HRAS	-0.684084464
ATF2	-0.683882651
BCL6	-0.606589293
HDAC4	-0.605771718

Table S6. Fold changes among differentially expressed genes for the 30 µg/mL *M. vaccae* NCTC 11659/RPMI 1640 vs. BBS/RPMI 1640 comparison in *Experiment 1* using the NanoString platform.

Gene symbol	log2FoldChange
Upregulated	
CCL8	3.65093055
CCL13	3.149080734
CCR7	3.042399552
IFIT3	2.878938362
MMP9	2.597995121
C1S	2.485042688
CXCL10	2.453873064
CXCL5	2.400323163
CCL22	2.334238711
ELK1	2.299251109
TRADD	2.036206871
CFB	2.00679544
IL8	1.533885836
PTGER2	1.46347844
HLA-DRB1	1.311637521
IL15	1.239788697
ITGB2	1.215136222
IFIT2	1.175723117
MRC1	1.123893462
IL11	1.110389565
CCL5	1.079814026
HMGN1	1.078072869
MAP3K7	1.060341309
PTGIR	1.052475684
NOD2	0.960361151
CD4	0.957328779
NLRP3	0.933533412
IL23A	0.933022695
RAF1	0.912271608
CXCL1	0.87168337
RELB	0.865295586
C1QB	0.843103513
OASL	0.840573093
CSF3	0.832248568

DEFA1	0.827830768
AREG	0.78106527
CCL24	0.760030653
HSPB1	0.752750259
SMAD7	0.750823952
CFL1	0.703655717
TREM2	0.699869662
CCR1	0.685080707
RHOA	0.672560739
MEF2D	0.670231674
CXCL2	0.659513981
Downregulated	
AGER	-5.482472487
OXER1	-3.18713
TLR7	-2.608692744
TNFSF14	-2.447947778
C5	-2.337656428
FXYD2	-2.046789727
PPP1R12B	-1.874183353
PTGS1	-1.644031939
RPS6KA5	-1.501620465
BIRC2	-1.493633999
PDGFA	-1.450070059
LTB4R2	-1.343424763
GRB2	-1.239103301
PTGS2	-1.18047607
IFIT1	-1.070359504
CEBPB	-1.059009382
LTB4R	-1.03310146
TGFB2	-1.004050778
LIMK1	-1.001134201
PRKCA	-0.999303406
IL23R	-0.957511838
CCR2	-0.933749827
FOS	-0.909354375
LTB	-0.89069282
HMGB1	-0.729197953
TYROBP	-0.634158315
PLA2G4A	-0.623782522

GNAS	-0.617431352
NOD1	-0.608089594
PLCB1	-0.590900884

Table S7. Fold changes among differentially expressed genes for the 30 μg/mL *M. vaccae* NCTC 11659/250 ng/mL LPS vs. BBS/250 ng/mL LPS comparison in *Experiment 1* using the NanoString platform.

Gene symbol	log2FoldChange
Upregulated	
TLR5	2.710084931
C1QA	2.684485845
LTB4R2	2.645940505
CXCR2	2.62672939
MAP2K6	2.453411249
CCR2	2.261744964
NOD1	1.963723256
FXYD2	1.582344947
CCL24	1.563842348
MRC1	1.537153018
TGFB2	1.50602263
C5	1.461106615
PRKCA	1.367938667
MAPK8	1.366182804
TNFSF14	1.34746473
HRAS	1.219384699
LTB4R	1.187643573
RPS6KA5	1.100961863
MAP3K5	1.033791185
CCL23	1.009685291
AREG	0.918382435
PPP1R12B	0.734176045
MAP2K4	0.703026041
NOD2	0.702369056
CCL21	0.667698549
PTGS2	0.624099384
Downregulated	
OASL	-2.784240439
IFIT1	-2.587393775
MX1	-2.33527973
CXCL10	-2.175247988
IFIT3	-2.05367924
CCR7	-1.728967684
CXCL5	-1.273190751

CXCL6	-1.09333728
CCL2	-1.093121135
CCL22	-0.989892313
STAT1	-0.965089457
CCL19	-0.906075234
IL1B	-0.853608949
AGER	-0.825973172
CCL20	-0.799298758
DEFA1	-0.73095191
TNFAIP3	-0.721129743
CCL4	-0.714245346
PTGER2	-0.71408325
PTGIR	-0.687632091
C1S	-0.672161381
CCL8	-0.59167732

Table S8. Fold changes among differentially expressed genes for the 30 μg/mL *M. vaccae* NCTC 11659/250 ng/mL LPS vs. 30 μg/mL *M. vaccae* NCTC 11659/RPMI 1640 comparison in *Experiment 1* using the NanoString platform.

Gene symbol	log2FoldChange
Upregulated	
LTB4R2	4.23281562
CXCR2	4.031971691
AGER	4.016773163
OXER1	4.009490272
FXYD2	4.008614412
C1QA	3.942186977
NOD1	3.811969852
RPS6KA5	3.79423271
TGFB2	3.736054254
TNFSF14	3.674022864
MAP2K6	3.55930419
C5	3.457229883
TLR5	3.456453149
CCR2	2.944770051
IL8	2.521621244
LTB	2.41142563
LTB4R	2.364887827
PRKCA	2.297443796
PPP1R12B	2.172234875
PTGS2	2.134592857
OAS2	2.125492439
MX2	2.111362529
IL1B	2.063350601
STAT1	1.999518899
CCL5	1.995405724
TLR7	1.929568056
CXCL3	1.824985615
CEBPB	1.699492904
MAP3K5	1.638747124
MAPK8	1.593596142
CCL23	1.497632409
MAP2K4	1.448478003
STAT2	1.375884108
CXCL2	1.361997517

PDGFA	1.324831159
CCL4	1.283845327
CCL24	1.138758278
IFIT1	1.089278947
CXCL1	1.007185964
CCL2	0.998759158
IL23R	0.957511838
IRF3	0.955314915
MRC1	0.880993491
CD40	0.839182724
IFI44	0.831107212
RIPK2	0.810193975
GRB2	0.735334921
TLR6	0.71876881
HRAS	0.679538836
CCL3	0.662215323
MX1	0.65700868
IL1RN	0.604535138
TLR8	0.603566607
NFKB1	0.592622323
PLCB1	0.590900884
Downregulated	
CCL13	-3.963713914
CCL8	-3.957226863
CXCL5	-3.510723636
C1S	-3.310503539
IFIT3	-3.03052879
CCR7	-2.538970211
PTGER2	-2.457891183
TREM2	-2.177287723
ELK1	-1.92381998
CFB	-1.888835541
CCL22	-1.887341581
IL1R1	-1.870613946
PTGIR	-1.836531367
OASL	-1.821113402
IL15	-1.766736742
MEF2D	-1.706026107
HMGN1	-1.693181947

CXCL10	-1.681440832
HLA-DRB1	-1.611129159
DEFA1	-1.608033126
IL11	-1.587978562
ITGB2	-1.565214359
CD4	-1.544090696
HSPB1	-1.395500204
MAP3K7	-1.37686946
TRADD	-1.293570771
MAPK14	-1.210057281
NLRP3	-1.202199016
CSF3	-1.197675934
ROCK2	-1.177821221
CFL1	-1.17165777
MAX	-1.033450101
IRF1	-0.971790096
MAPKAPK2	-0.962311663
NR3C1	-0.959382127
TGFB1	-0.940185258
CCR1	-0.92268522
C1QB	-0.843103513
STAT3	-0.832116767
GNAS	-0.791271867
GNAQ	-0.77341131
LY96	-0.771267718
RHOA	-0.76971607
RAF1	-0.769392504
BCL6	-0.767311415
MMP9	-0.762833124
CREB1	-0.73924081
HMGB2	-0.709822123
SMAD7	-0.676374314
TRAF2	-0.6481829
TNF	-0.62012128

Table S9. Sample sizes (*n*) for *Experiment 2* consisting of real time RT-PCR experiments examining the effect of LPS and *M. vaccae* NCTC 11659 on gene expression. Sample size represents 1 experimental replicate. Data came from two independent experiments (*N* = 2).

Figure	Gene	LPS treatment	Bacterial Treatment	Sample Size (*n*)
S13	*IL12A*	0 ng/mL	BBS	2
			10 µg/mL *M. vaccae* NCTC 11659	1
			30 µg/mL *M. vaccae* NCTC 11659	4
			100 µg/mL *M. vaccae* NCTC 11659	4
			300 µg/mL *M. vaccae* NCTC 11659	2
		0.5 ng/mL	BBS	7
			10 µg/mL *M. vaccae* NCTC 11659	8
			30 µg/mL *M. vaccae* NCTC 11659	8
			100 µg/mL *M. vaccae* NCTC 11659	8
			300 µg/mL *M. vaccae* NCTC 11659	8
		2.5 ng/mL	BBS	7
			10 µg/mL *M. vaccae* NCTC 11659	7
			30 µg/mL *M. vaccae* NCTC 11659	8
			100 µg/mL *M. vaccae* NCTC 11659	8
			300 µg/mL *M. vaccae* NCTC 11659	7
		250 ng/mL	BBS	4
			10 µg/mL *M. vaccae* NCTC 11659	8
			30 µg/mL *M. vaccae* NCTC 11659	7
			100 µg/mL *M. vaccae* NCTC 11659	8
			300 µg/mL *M. vaccae* NCTC 11659	7
	IL12B	0 ng/mL	BBS	3
			10 µg/mL *M. vaccae* NCTC 11659	3
			30 µg/mL *M. vaccae* NCTC 11659	5
			100 µg/mL *M. vaccae* NCTC 11659	8
			300 µg/mL *M. vaccae* NCTC 11659	5
		0.5 ng/mL	BBS	8
			10 µg/mL *M. vaccae* NCTC 11659	8
			30 µg/mL *M. vaccae* NCTC 11659	8
			100 µg/mL *M. vaccae* NCTC 11659	8
			300 µg/mL *M. vaccae* NCTC 11659	8
		2.5 ng/mL	BBS	7
			10 µg/mL *M. vaccae* NCTC 11659	8
			30 µg/mL *M. vaccae* NCTC 11659	8
			100 µg/mL *M. vaccae* NCTC 11659	8
			300 µg/mL *M. vaccae* NCTC 11659	8

		250 ng/mL	BBS	8
			10 µg/mL *M. vaccae* NCTC 11659	8
			30 µg/mL *M. vaccae* NCTC 11659	8
			100 µg/mL *M. vaccae* NCTC 11659	8
			300 µg/mL *M. vaccae* NCTC 11659	8
	IL23A	0 ng/mL	BBS	8
			10 µg/mL *M. vaccae* NCTC 11659	8
			30 µg/mL *M. vaccae* NCTC 11659	8
			100 µg/mL *M. vaccae* NCTC 11659	8
			300 µg/mL *M. vaccae* NCTC 11659	8
		0.5 ng/mL	BBS	8
			10 µg/mL *M. vaccae* NCTC 11659	8
			30 µg/mL *M. vaccae* NCTC 11659	8
			100 µg/mL *M. vaccae* NCTC 11659	8
			300 µg/mL *M. vaccae* NCTC 11659	8
		2.5 ng/mL	BBS	8
			10 µg/mL *M. vaccae* NCTC 11659	8
			30 µg/mL *M. vaccae* NCTC 11659	8
			100 µg/mL *M. vaccae* NCTC 11659	8
			300 µg/mL *M. vaccae* NCTC 11659	8
		250 ng/mL	BBS	8
			10 µg/mL *M. vaccae* NCTC 11659	8
			30 µg/mL *M. vaccae* NCTC 11659	8
			100 µg/mL *M. vaccae* NCTC 11659	8
			300 µg/mL *M. vaccae* NCTC 11659	8
S14	*IL10*	0 ng/mL	BBS	8
			10 µg/mL *M. vaccae* NCTC 11659	7
			30 µg/mL *M. vaccae* NCTC 11659	8
			100 µg/mL *M. vaccae* NCTC 11659	8
			300 µg/mL *M. vaccae* NCTC 11659	8
		0.5 ng/mL	BBS	8
			10 µg/mL *M. vaccae* NCTC 11659	8
			30 µg/mL *M. vaccae* NCTC 11659	8
			100 µg/mL *M. vaccae* NCTC 11659	8
			300 µg/mL *M. vaccae* NCTC 11659	8
		2.5 ng/mL	BBS	8
			10 µg/mL *M. vaccae* NCTC 11659	8
			30 µg/mL *M. vaccae* NCTC 11659	8
			100 µg/mL *M. vaccae* NCTC 11659	8

			300 μg/mL *M. vaccae* NCTC 11659	8
		250 ng/mL	BBS	8
			10 μg/mL *M. vaccae* NCTC 11659	8
			30 μg/mL *M. vaccae* NCTC 11659	8
			100 μg/mL *M. vaccae* NCTC 11659	8
			300 μg/mL *M. vaccae* NCTC 11659	8
	TGFB	0 ng/mL	BBS	8
			10 μg/mL *M. vaccae* NCTC 11659	7
			30 μg/mL *M. vaccae* NCTC 11659	8
			100 μg/mL *M. vaccae* NCTC 11659	8
			300 μg/mL *M. vaccae* NCTC 11659	8
		0.5 ng/mL	BBS	8
			10 μg/mL *M. vaccae* NCTC 11659	8
			30 μg/mL *M. vaccae* NCTC 11659	8
			100 μg/mL *M. vaccae* NCTC 11659	8
			300 μg/mL *M. vaccae* NCTC 11659	8
		2.5 ng/mL	BBS	7
			10 μg/mL *M. vaccae* NCTC 11659	8
			30 μg/mL *M. vaccae* NCTC 11659	7
			100 μg/mL *M. vaccae* NCTC 11659	8
			300 μg/mL *M. vaccae* NCTC 11659	8
		250 ng/mL	BBS	8
			10 μg/mL *M. vaccae* NCTC 11659	8
			30 μg/mL *M. vaccae* NCTC 11659	8
			100 μg/mL *M. vaccae* NCTC 11659	8
			300 μg/mL *M. vaccae* NCTC 11659	8
2	*IL10:IL12A*	0 ng/mL	BBS	2
			10 μg/mL *M. vaccae* NCTC 11659	1
			30 μg/mL *M. vaccae* NCTC 11659	3
			100 μg/mL *M. vaccae* NCTC 11659	4
			300 μg/mL *M. vaccae* NCTC 11659	2
		0.5 ng/mL	BBS	7
			10 μg/mL *M. vaccae* NCTC 11659	8
			30 μg/mL *M. vaccae* NCTC 11659	8
			100 μg/mL *M. vaccae* NCTC 11659	8
			300 μg/mL *M. vaccae* NCTC 11659	8
		2.5 ng/mL	BBS	7
			10 μg/mL *M. vaccae* NCTC 11659	7
			30 μg/mL *M. vaccae* NCTC 11659	8

			100 µg/mL *M. vaccae* NCTC 11659	8
			300 µg/mL *M. vaccae* NCTC 11659	7
		250 ng/mL	BBS	4
			10 µg/mL *M. vaccae* NCTC 11659	8
			30 µg/mL *M. vaccae* NCTC 11659	7
			100 µg/mL *M. vaccae* NCTC 11659	8
			300 µg/mL *M. vaccae* NCTC 11659	7
	IL10:IL12B	0 ng/mL	BBS	3
			10 µg/mL *M. vaccae* NCTC 11659	2
			30 µg/mL *M. vaccae* NCTC 11659	5
			100 µg/mL *M. vaccae* NCTC 11659	8
			300 µg/mL *M. vaccae* NCTC 11659	4
		0.5 ng/mL	BBS	8
			10 µg/mL *M. vaccae* NCTC 11659	8
			30 µg/mL *M. vaccae* NCTC 11659	8
			100 µg/mL *M. vaccae* NCTC 11659	8
			300 µg/mL *M. vaccae* NCTC 11659	8
		2.5 ng/mL	BBS	8
			10 µg/mL *M. vaccae* NCTC 11659	8
			30 µg/mL *M. vaccae* NCTC 11659	8
			100 µg/mL *M. vaccae* NCTC 11659	8
			300 µg/mL *M. vaccae* NCTC 11659	7
		250 ng/mL	BBS	7
			10 µg/mL *M. vaccae* NCTC 11659	8
			30 µg/mL *M. vaccae* NCTC 11659	8
			100 µg/mL *M. vaccae* NCTC 11659	8
			300 µg/mL *M. vaccae* NCTC 11659	8
	IL10:IL23A	0 ng/mL	BBS	8
			10 µg/mL *M. vaccae* NCTC 11659	7
			30 µg/mL *M. vaccae* NCTC 11659	8
			100 µg/mL *M. vaccae* NCTC 11659	8
			300 µg/mL *M. vaccae* NCTC 11659	8
		0.5 ng/mL	BBS	8
			10 µg/mL *M. vaccae* NCTC 11659	8
			30 µg/mL *M. vaccae* NCTC 11659	8
			100 µg/mL *M. vaccae* NCTC 11659	8
			300 µg/mL *M. vaccae* NCTC 11659	8
		2.5 ng/mL	BBS	8
			10 µg/mL *M. vaccae* NCTC 11659	8

			30 µg/mL *M. vaccae* NCTC 11659	8
			100 µg/mL *M. vaccae* NCTC 11659	8
			300 µg/mL *M. vaccae* NCTC 11659	8
		250 ng/mL	BBS	8
			10 µg/mL *M. vaccae* NCTC 11659	8
			30 µg/mL *M. vaccae* NCTC 11659	8
			100 µg/mL *M. vaccae* NCTC 11659	8
			300 µg/mL *M. vaccae* NCTC 11659	8
S15	*TGFB:IL12A*	0 ng/mL	BBS	2
			10 µg/mL *M. vaccae* NCTC 11659	1
			30 µg/mL *M. vaccae* NCTC 11659	4
			100 µg/mL *M. vaccae* NCTC 11659	4
			300 µg/mL *M. vaccae* NCTC 11659	2
		0.5 ng/mL	BBS	7
			10 µg/mL *M. vaccae* NCTC 11659	8
			30 µg/mL *M. vaccae* NCTC 11659	8
			100 µg/mL *M. vaccae* NCTC 11659	8
			300 µg/mL *M. vaccae* NCTC 11659	8
		2.5 ng/mL	BBS	7
			10 µg/mL *M. vaccae* NCTC 11659	7
			30 µg/mL *M. vaccae* NCTC 11659	7
			100 µg/mL *M. vaccae* NCTC 11659	8
			300 µg/mL *M. vaccae* NCTC 11659	7
		250 ng/mL	BBS	4
			10 µg/mL *M. vaccae* NCTC 11659	8
			30 µg/mL *M. vaccae* NCTC 11659	7
			100 µg/mL *M. vaccae* NCTC 11659	8
			300 µg/mL *M. vaccae* NCTC 11659	7
	TGFB:IL12B	0 ng/mL	BBS	3
			10 µg/mL *M. vaccae* NCTC 11659	3
			30 µg/mL *M. vaccae* NCTC 11659	5
			100 µg/mL *M. vaccae* NCTC 11659	8
			300 µg/mL *M. vaccae* NCTC 11659	4
		0.5 ng/mL	BBS	8
			10 µg/mL *M. vaccae* NCTC 11659	8
			30 µg/mL *M. vaccae* NCTC 11659	8
			100 µg/mL *M. vaccae* NCTC 11659	8
			300 µg/mL *M. vaccae* NCTC 11659	8
		2.5 ng/mL	BBS	6

			10 µg/mL *M. vaccae* NCTC 11659	8
			30 µg/mL *M. vaccae* NCTC 11659	7
			100 µg/mL *M. vaccae* NCTC 11659	7
			300 µg/mL *M. vaccae* NCTC 11659	8
		250 ng/mL	BBS	8
			10 µg/mL *M. vaccae* NCTC 11659	8
			30 µg/mL *M. vaccae* NCTC 11659	8
			100 µg/mL *M. vaccae* NCTC 11659	8
			300 µg/mL *M. vaccae* NCTC 11659	8
	TGFB:IL23A	0 ng/mL	BBS	8
			10 µg/mL *M. vaccae* NCTC 11659	8
			30 µg/mL *M. vaccae* NCTC 11659	8
			100 µg/mL *M. vaccae* NCTC 11659	8
			300 µg/mL *M. vaccae* NCTC 11659	8
		0.5 ng/mL	BBS	8
			10 µg/mL *M. vaccae* NCTC 11659	8
			30 µg/mL *M. vaccae* NCTC 11659	8
			100 µg/mL *M. vaccae* NCTC 11659	8
			300 µg/mL *M. vaccae* NCTC 11659	8
		2.5 ng/mL	BBS	7
			10 µg/mL *M. vaccae* NCTC 11659	8
			30 µg/mL *M. vaccae* NCTC 11659	7
			100 µg/mL *M. vaccae* NCTC 11659	8
			300 µg/mL *M. vaccae* NCTC 11659	8
		250 ng/mL	BBS	8
			10 µg/mL *M. vaccae* NCTC 11659	8
			30 µg/mL *M. vaccae* NCTC 11659	8
			100 µg/mL *M. vaccae* NCTC 11659	8
			300 µg/mL *M. vaccae* NCTC 11659	8

Table S10. Post hoc *p* values not specified in main text.

Figure	Gene	LPS condition	Comparison	*p*
S13	*IL12A*	Within 0 ng/mL	BBS vs. 10 µg/mL *M. vaccae* NCTC 11659	> .1
			BBS vs. 30 µg/mL *M. vaccae* NCTC 11659	> .1
			BBS vs. 100 µg/mL *M. vaccae* NCTC 11659	> .1
			BBS vs. 300 µg/mL *M. vaccae* NCTC 11659	> .1
		Within 0.5 ng/mL	BBS vs. 10 µg/mL *M. vaccae* NCTC 11659	> .1
			BBS vs. 30 µg/mL *M. vaccae* NCTC 11659	> .1
			BBS vs. 100 µg/mL *M. vaccae* NCTC 11659	> .1
			BBS vs. 300 µg/mL *M. vaccae* NCTC 11659	> .1
		Within 2.5 ng/mL	BBS vs. 10 µg/mL *M. vaccae* NCTC 11659	> .1
			BBS vs. 30 µg/mL *M. vaccae* NCTC 11659	> .1
			BBS vs. 100 µg/mL *M. vaccae* NCTC 11659	> .1
			BBS vs. 300 µg/mL *M. vaccae* NCTC 11659	0.0688819
		Within 250 ng/mL	BBS vs. 10 µg/mL *M. vaccae* NCTC 11659	> .1
			BBS vs. 30 µg/mL *M. vaccae* NCTC 11659	> .1
			BBS vs. 100 µg/mL *M. vaccae* NCTC 11659	> .1
			BBS vs. 300 µg/mL *M. vaccae* NCTC 11659	> .1
		Between all LPS conditions within BBS	BBS + LPS vehicle vs. BBS + 0.5 ng/mL LPS	> .1
			BBS + LPS vehicle vs. BBS + 2.5 ng/mL LPS	> .1
			BBS + LPS vehicle vs. BBS + 250 ng/mL LPS	> .1
		Between all LPS conditions within 10 µg/mL *M. vaccae* NCTC 11659	10 µg/mL *M. vaccae* NCTC 11659 + LPS vehicle vs. 10 µg/mL *M. vaccae* NCTC 11659 + 0.5 ng/mL LPS	> .1

			10 µg/mL *M. vaccae* NCTC 11659 + LPS vehicle vs. 10 µg/mL *M. vaccae* NCTC 11659 + 2.5 ng/mL LPS	> .1
			10 µg/mL *M. vaccae* NCTC 11659 + LPS vehicle vs. 10 µg/mL *M. vaccae* NCTC 11659 + 250 ng/mL LPS	0.01458014
		Between all LPS conditions within 30 µg/mL *M. vaccae* NCTC 11659	30 µg/mL *M. vaccae* NCTC 11659 + LPS vehicle vs. 30 µg/mL *M. vaccae* NCTC 11659 + 0.5 ng/mL LPS	> .1
			30 µg/mL *M. vaccae* NCTC 11659 + LPS vehicle vs. 30 µg/mL *M. vaccae* NCTC 11659 + 2.5 ng/mL LPS	> .1
			30 µg/mL *M. vaccae* NCTC 11659 + LPS vehicle vs. 30 µg/mL *M. vaccae* NCTC 11659 + 250 ng/mL LPS	> .1
		Between all LPS conditions within 100 µg/mL *M. vaccae* NCTC 11659	100 µg/mL *M. vaccae* NCTC 11659 + LPS vehicle vs. 100 µg/mL *M. vaccae* NCTC 11659 + 0.5 ng/mL LPS	> .1
			100 µg/mL *M. vaccae* NCTC 11659 + LPS vehicle vs. 100 µg/mL *M. vaccae* NCTC 11659 + 2.5 ng/mL LPS	> .1
			100 µg/mL *M. vaccae* NCTC 11659 + LPS vehicle vs. 100 µg/mL *M. vaccae* NCTC 11659 + 250 ng/mL LPS	> .1
		Between all LPS conditions within 300 µg/mL *M. vaccae* NCTC 11659	300 µg/mL *M. vaccae* NCTC 11659 + LPS vehicle vs. 300 µg/mL *M. vaccae* NCTC 11659 + 0.5 ng/mL LPS	> .1
			300 µg/mL *M. vaccae* NCTC 11659 + LPS vehicle vs. 300 µg/mL *M. vaccae* NCTC 11659 + 2.5 ng/mL LPS	> .1
			300 µg/mL *M. vaccae* NCTC 11659 + LPS vehicle vs. 300 µg/mL *M. vaccae* NCTC 11659 + 250 ng/mL LPS	0.01240536
	IL12B	Within 0 ng/mL	BBS vs. 10 µg/mL *M. vaccae* NCTC 11659	> .1

			BBS vs. 30 µg/mL *M. vaccae* NCTC 11659	< .01
			BBS vs. 100 µg/mL *M. vaccae* NCTC 11659	< .01
			BBS vs. 300 µg/mL *M. vaccae* NCTC 11659	< .01
		Within 0.5 ng/mL	BBS vs. 10 µg/mL *M. vaccae* NCTC 11659	> .1
			BBS vs. 30 µg/mL *M. vaccae* NCTC 11659	> .1
			BBS vs. 100 µg/mL *M. vaccae* NCTC 11659	< .01
			BBS vs. 300 µg/mL *M. vaccae* NCTC 11659	< .01
		Within 2.5 ng/mL	BBS vs. 10 µg/mL *M. vaccae* NCTC 11659	> .1
			BBS vs. 30 µg/mL *M. vaccae* NCTC 11659	0.0789984
			BBS vs. 100 µg/mL *M. vaccae* NCTC 11659	0.09231063
			BBS vs. 300 µg/mL *M. vaccae* NCTC 11659	> .1
		Within 250 ng/mL	BBS vs. 10 µg/mL *M. vaccae* NCTC 11659	> .1
			BBS vs. 30 µg/mL *M. vaccae* NCTC 11659	> .1
			BBS vs. 100 µg/mL *M. vaccae* NCTC 11659	0.01916455
			BBS vs. 300 µg/mL *M. vaccae* NCTC 11659	< .01
		Between all LPS conditions within BBS	BBS + LPS vehicle vs. BBS + 0.5 ng/mL LPS	< .01
			BBS + LPS vehicle vs. BBS + 2.5 ng/mL LPS	< .001
			BBS + LPS vehicle vs. BBS + 250 ng/mL LPS	< .001
		Between all LPS conditions within 10 µg/mL *M. vaccae* NCTC 11659	10 µg/mL *M. vaccae* NCTC 11659 + LPS vehicle vs. 10 µg/mL *M. vaccae* NCTC 11659 + 0.5 ng/mL LPS	> .1
			10 µg/mL *M. vaccae* NCTC 11659 + LPS vehicle vs. 10 µg/mL *M. vaccae* NCTC 11659 + 2.5 ng/mL LPS	< .01

			10 µg/mL *M. vaccae* NCTC 11659 + LPS vehicle vs. 10 µg/mL *M. vaccae* NCTC 11659 + 250 ng/mL LPS	< .001
		Between all LPS conditions within 30 µg/mL *M. vaccae* NCTC 11659	30 µg/mL *M. vaccae* NCTC 11659 + LPS vehicle vs. 30 µg/mL *M. vaccae* NCTC 11659 + 0.5 ng/mL LPS	> .1
			30 µg/mL *M. vaccae* NCTC 11659 + LPS vehicle vs. 30 µg/mL *M. vaccae* NCTC 11659 + 2.5 ng/mL LPS	> .1
			30 µg/mL *M. vaccae* NCTC 11659 + LPS vehicle vs. 30 µg/mL *M. vaccae* NCTC 11659 + 250 ng/mL LPS	< .001
		Between all LPS conditions within 100 µg/mL *M. vaccae* NCTC 11659	100 µg/mL *M. vaccae* NCTC 11659 + LPS vehicle vs. 100 µg/mL *M. vaccae* NCTC 11659 + 0.5 ng/mL LPS	> .1
			100 µg/mL *M. vaccae* NCTC 11659 + LPS vehicle vs. 100 µg/mL *M. vaccae* NCTC 11659 + 2.5 ng/mL LPS	> .1
			100 µg/mL *M. vaccae* NCTC 11659 + LPS vehicle vs. 100 µg/mL *M. vaccae* NCTC 11659 + 250 ng/mL LPS	< .001
		Between all LPS conditions within 300 µg/mL *M. vaccae* NCTC 11659	300 µg/mL *M. vaccae* NCTC 11659 + LPS vehicle vs. 300 µg/mL *M. vaccae* NCTC 11659 + 0.5 ng/mL LPS	> .1
			300 µg/mL *M. vaccae* NCTC 11659 + LPS vehicle vs. 300 µg/mL *M. vaccae* NCTC 11659 + 2.5 ng/mL LPS	> .1
			300 µg/mL *M. vaccae* NCTC 11659 + LPS vehicle vs. 300 µg/mL *M. vaccae* NCTC 11659 + 250 ng/mL LPS	< .001
	IL23A	Within 0 ng/mL	BBS vs. 10 µg/mL *M. vaccae* NCTC 11659	< .01
			BBS vs. 30 µg/mL *M. vaccae* NCTC 11659	< .01
			BBS vs. 100 µg/mL *M. vaccae* NCTC 11659	< .001

			BBS vs. 300 µg/mL *M. vaccae* NCTC 11659	< .001
		Within 0.5 ng/mL	BBS vs. 10 µg/mL *M. vaccae* NCTC 11659	> .1
			BBS vs. 30 µg/mL *M. vaccae* NCTC 11659	> .1
			BBS vs. 100 µg/mL *M. vaccae* NCTC 11659	> .1
			BBS vs. 300 µg/mL *M. vaccae* NCTC 11659	> .1
		Within 2.5 ng/mL	BBS vs. 10 µg/mL *M. vaccae* NCTC 11659	> .1
			BBS vs. 30 µg/mL *M. vaccae* NCTC 11659	> .1
			BBS vs. 100 µg/mL *M. vaccae* NCTC 11659	> .1
			BBS vs. 300 µg/mL *M. vaccae* NCTC 11659	> .1
		Within 250 ng/mL	BBS vs. 10 µg/mL *M. vaccae* NCTC 11659	> .1
			BBS vs. 30 µg/mL *M. vaccae* NCTC 11659	> .1
			BBS vs. 100 µg/mL *M. vaccae* NCTC 11659	< .01
			BBS vs. 300 µg/mL *M. vaccae* NCTC 11659	< .01
		Between all LPS conditions within BBS	BBS + LPS vehicle vs. BBS + 0.5 ng/mL LPS	< .01
			BBS + LPS vehicle vs. BBS + 2.5 ng/mL LPS	< .001
			BBS + LPS vehicle vs. BBS + 250 ng/mL LPS	< .001
		Between all LPS conditions within 10 µg/mL *M. vaccae* NCTC 11659	10 µg/mL *M. vaccae* NCTC 11659 + LPS vehicle vs. 10 µg/mL *M. vaccae* NCTC 11659 + 0.5 ng/mL LPS	> .1
			10 µg/mL *M. vaccae* NCTC 11659 + LPS vehicle vs. 10 µg/mL *M. vaccae* NCTC 11659 + 2.5 ng/mL LPS	< .01
			10 µg/mL *M. vaccae* NCTC 11659 + LPS vehicle vs. 10 µg/mL *M. vaccae* NCTC 11659 + 250 ng/mL LPS	< .001

			30 µg/mL *M. vaccae* NCTC 11659 + LPS vehicle vs. 30 µg/mL *M. vaccae* NCTC 11659 + 0.5 ng/mL LPS	> .1
		Between all LPS conditions within 30 µg/mL *M. vaccae* NCTC 11659	30 µg/mL *M. vaccae* NCTC 11659 + LPS vehicle vs. 30 µg/mL *M. vaccae* NCTC 11659 + 2.5 ng/mL LPS	0.09659867
			30 µg/mL *M. vaccae* NCTC 11659 + LPS vehicle vs. 30 µg/mL *M. vaccae* NCTC 11659 + 250 ng/mL LPS	< .001
			100 µg/mL *M. vaccae* NCTC 11659 + LPS vehicle vs. 100 µg/mL *M. vaccae* NCTC 11659 + 0.5 ng/mL LPS	> .1
		Between all LPS conditions within 100 µg/mL *M. vaccae* NCTC 11659	100 µg/mL *M. vaccae* NCTC 11659 + LPS vehicle vs. 100 µg/mL *M. vaccae* NCTC 11659 + 2.5 ng/mL LPS	> .1
			100 µg/mL *M. vaccae* NCTC 11659 + LPS vehicle vs. 100 µg/mL *M. vaccae* NCTC 11659 + 250 ng/mL LPS	< .001
			300 µg/mL *M. vaccae* NCTC 11659 + LPS vehicle vs. 300 µg/mL *M. vaccae* NCTC 11659 + 0.5 ng/mL LPS	< .01
		Between all LPS conditions within 300 µg/mL *M. vaccae* NCTC 11659	300 µg/mL *M. vaccae* NCTC 11659 + LPS vehicle vs. 300 µg/mL *M. vaccae* NCTC 11659 + 2.5 ng/mL LPS	> .1
			300 µg/mL *M. vaccae* NCTC 11659 + LPS vehicle vs. 300 µg/mL *M. vaccae* NCTC 11659 + 250 ng/mL LPS	< .01
S14	*IL10*		BBS vs. 10 µg/mL *M. vaccae* NCTC 11659	0.03136868
		Within 0 ng/mL	BBS vs. 30 µg/mL *M. vaccae* NCTC 11659	0.0282815
			BBS vs. 100 µg/mL *M. vaccae* NCTC 11659	0.03863024
			BBS vs. 300 µg/mL *M. vaccae* NCTC 11659	> .1
		Within 0.5 ng/mL	BBS vs. 10 µg/mL *M. vaccae* NCTC 11659	> .1

			BBS vs. 30 µg/mL *M. vaccae* NCTC 11659	> .1
			BBS vs. 100 µg/mL *M. vaccae* NCTC 11659	0.02616086
			BBS vs. 300 µg/mL *M. vaccae* NCTC 11659	< .01
		Within 2.5 ng/mL	BBS vs. 10 µg/mL *M. vaccae* NCTC 11659	> .1
			BBS vs. 30 µg/mL *M. vaccae* NCTC 11659	> .1
			BBS vs. 100 µg/mL *M. vaccae* NCTC 11659	0.04470415
			BBS vs. 300 µg/mL *M. vaccae* NCTC 11659	< .01
		Within 250 ng/mL	BBS vs. 10 µg/mL *M. vaccae* NCTC 11659	> .1
			BBS vs. 30 µg/mL *M. vaccae* NCTC 11659	> .1
			BBS vs. 100 µg/mL *M. vaccae* NCTC 11659	> .1
			BBS vs. 300 µg/mL *M. vaccae* NCTC 11659	> .1
		Between all LPS conditions within BBS	BBS + LPS vehicle vs. BBS + 0.5 ng/mL LPS	0.0232713
			BBS + LPS vehicle vs. BBS + 2.5 ng/mL LPS	< .01
			BBS + LPS vehicle vs. BBS + 250 ng/mL LPS	> .1
		Between all LPS conditions within 10 µg/mL *M. vaccae* NCTC 11659	10 µg/mL *M. vaccae* NCTC 11659 + LPS vehicle vs. 10 µg/mL *M. vaccae* NCTC 11659 + 0.5 ng/mL LPS	> .1
			10 µg/mL *M. vaccae* NCTC 11659 + LPS vehicle vs. 10 µg/mL *M. vaccae* NCTC 11659 + 2.5 ng/mL LPS	> .1
			10 µg/mL *M. vaccae* NCTC 11659 + LPS vehicle vs. 10 µg/mL *M. vaccae* NCTC 11659 + 250 ng/mL LPS	> .1
		Between all LPS conditions within 30 µg/mL *M. vaccae* NCTC 11659	30 µg/mL *M. vaccae* NCTC 11659 + LPS vehicle vs. 30 µg/mL *M. vaccae* NCTC 11659 + 0.5 ng/mL LPS	> .1

			30 µg/mL *M. vaccae* NCTC 11659 + LPS vehicle vs. 30 µg/mL *M. vaccae* NCTC 11659 + 2.5 ng/mL LPS	> .1
			30 µg/mL *M. vaccae* NCTC 11659 + LPS vehicle vs. 30 µg/mL *M. vaccae* NCTC 11659 + 250 ng/mL LPS	> .1
		Between all LPS conditions within 100 µg/mL *M. vaccae* NCTC 11659	100 µg/mL *M. vaccae* NCTC 11659 + LPS vehicle vs. 100 µg/mL *M. vaccae* NCTC 11659 + 0.5 ng/mL LPS	0.03190491
			100 µg/mL *M. vaccae* NCTC 11659 + LPS vehicle vs. 100 µg/mL *M. vaccae* NCTC 11659 + 2.5 ng/mL LPS	> .1
			100 µg/mL *M. vaccae* NCTC 11659 + LPS vehicle vs. 100 µg/mL *M. vaccae* NCTC 11659 + 250 ng/mL LPS	> .1
		Between all LPS conditions within 300 µg/mL *M. vaccae* NCTC 11659	300 µg/mL *M. vaccae* NCTC 11659 + LPS vehicle vs. 300 µg/mL *M. vaccae* NCTC 11659 + 0.5 ng/mL LPS	< .01
			300 µg/mL *M. vaccae* NCTC 11659 + LPS vehicle vs. 300 µg/mL *M. vaccae* NCTC 11659 + 2.5 ng/mL LPS	0.03972336
			300 µg/mL *M. vaccae* NCTC 11659 + LPS vehicle vs. 300 µg/mL *M. vaccae* NCTC 11659 + 250 ng/mL LPS	0.0101786
	TGFB	Within 0 ng/mL	BBS vs. 10 µg/mL *M. vaccae* NCTC 11659	> .1
			BBS vs. 30 µg/mL *M. vaccae* NCTC 11659	> .1
			BBS vs. 100 µg/mL *M. vaccae* NCTC 11659	> .1
			BBS vs. 300 µg/mL *M. vaccae* NCTC 11659	> .1
		Within 0.5 ng/mL	BBS vs. 10 µg/mL *M. vaccae* NCTC 11659	> .1
			BBS vs. 30 µg/mL *M. vaccae* NCTC 11659	> .1
			BBS vs. 100 µg/mL *M. vaccae* NCTC 11659	> .1

			BBS vs. 300 µg/mL *M. vaccae* NCTC 11659	> .1
		Within 250 ng/mL	BBS vs. 10 µg/mL *M. vaccae* NCTC 11659	> .1
			BBS vs. 30 µg/mL *M. vaccae* NCTC 11659	> .1
			BBS vs. 100 µg/mL *M. vaccae* NCTC 11659	> .1
			BBS vs. 300 µg/mL *M. vaccae* NCTC 11659	> .1
		Within 2.5 ng/mL	BBS vs. 10 µg/mL *M. vaccae* NCTC 11659	> .1
			BBS vs. 30 µg/mL *M. vaccae* NCTC 11659	0.03918828
			BBS vs. 100 µg/mL *M. vaccae* NCTC 11659	0.06495136
			BBS vs. 300 µg/mL *M. vaccae* NCTC 11659	0.03227054
		Between all LPS conditions within BBS	BBS + LPS vehicle vs. BBS + 0.5 ng/mL LPS	> .1
			BBS + LPS vehicle vs. BBS + 250 ng/mL LPS	> .1
			BBS + LPS vehicle vs. BBS + 2.5 ng/mL LPS	0.0433803
		Between all LPS conditions within 10 µg/mL *M. vaccae* NCTC 11659	10 µg/mL *M. vaccae* NCTC 11659 + LPS vehicle vs. 10 µg/mL *M. vaccae* NCTC 11659 + 0.5 ng/mL LPS	> .1
			10 µg/mL *M. vaccae* NCTC 11659 + LPS vehicle vs. 10 µg/mL *M. vaccae* NCTC 11659 + 250 ng/mL LPS	> .1
			10 µg/mL *M. vaccae* NCTC 11659 + LPS vehicle vs. 10 µg/mL *M. vaccae* NCTC 11659 + 2.5 ng/mL LPS	0.01099135
		Between all LPS conditions within 30 µg/mL *M. vaccae* NCTC 11659	30 µg/mL *M. vaccae* NCTC 11659 + LPS vehicle vs. 30 µg/mL *M. vaccae* NCTC 11659 + 0.5 ng/mL LPS	> .1
			30 µg/mL *M. vaccae* NCTC 11659 + LPS vehicle vs. 30 µg/mL *M. vaccae* NCTC 11659 + 250 ng/mL LPS	0.01357702

			30 µg/mL *M. vaccae* NCTC 11659 + LPS vehicle vs. 30 µg/mL *M. vaccae* NCTC 11659 + 2.5 ng/mL LPS	< .001
		Between all LPS conditions within 100 µg/mL *M. vaccae* NCTC 11659	100 µg/mL *M. vaccae* NCTC 11659 + LPS vehicle vs. 100 µg/mL *M. vaccae* NCTC 11659 + 0.5 ng/mL LPS	> .1
			100 µg/mL *M. vaccae* NCTC 11659 + LPS vehicle vs. 100 µg/mL *M. vaccae* NCTC 11659 + 2.5 ng/mL LPS	> .1
			100 µg/mL *M. vaccae* NCTC 11659 + LPS vehicle vs. 100 µg/mL *M. vaccae* NCTC 11659 + 250 ng/mL LPS	> .1
		Between all LPS conditions within 300 µg/mL *M. vaccae* NCTC 11659	300 µg/mL *M. vaccae* NCTC 11659 + LPS vehicle vs. 300 µg/mL *M. vaccae* NCTC 11659 + 0.5 ng/mL LPS	> .1
			300 µg/mL *M. vaccae* NCTC 11659 + LPS vehicle vs. 300 µg/mL *M. vaccae* NCTC 11659 + 2.5 ng/mL LPS	> .1
			300 µg/mL *M. vaccae* NCTC 11659 + LPS vehicle vs. 300 µg/mL *M. vaccae* NCTC 11659 + 250 ng/mL LPS	> .1
2	*IL10:IL12A*	Within 0 ng/mL	BBS vs. 10 µg/mL *M. vaccae* NCTC 11659	> .1
			BBS vs. 30 µg/mL *M. vaccae* NCTC 11659	> .1
			BBS vs. 100 µg/mL *M. vaccae* NCTC 11659	> .1
			BBS vs. 300 µg/mL *M. vaccae* NCTC 11659	> .1
		Within 0.5 ng/mL	BBS vs. 10 µg/mL *M. vaccae* NCTC 11659	> .1
			BBS vs. 30 µg/mL *M. vaccae* NCTC 11659	> .1
			BBS vs. 100 µg/mL *M. vaccae* NCTC 11659	0.01217065
			BBS vs. 300 µg/mL *M. vaccae* NCTC 11659	0.0264807
		Within 2.5 ng/mL	BBS vs. 10 µg/mL *M. vaccae* NCTC 11659	> .1

			BBS vs. 30 µg/mL *M. vaccae* NCTC 11659	> .1
			BBS vs. 100 µg/mL *M. vaccae* NCTC 11659	> .1
			BBS vs. 300 µg/mL *M. vaccae* NCTC 11659	> .1
		Within 250 ng/mL	BBS vs. 10 µg/mL *M. vaccae* NCTC 11659	> .1
			BBS vs. 30 µg/mL *M. vaccae* NCTC 11659	> .1
			BBS vs. 100 µg/mL *M. vaccae* NCTC 11659	> .1
			BBS vs. 300 µg/mL *M. vaccae* NCTC 11659	> .1
		Between all LPS conditions within BBS	BBS + LPS vehicle vs. BBS + 0.5 ng/mL LPS	0.06869352
			BBS + LPS vehicle vs. BBS + 2.5 ng/mL LPS	> .1
			BBS + LPS vehicle vs. BBS + 250 ng/mL LPS	> .1
		Between all LPS conditions within 10 µg/mL *M. vaccae* NCTC 11659	10 µg/mL *M. vaccae* NCTC 11659 + LPS vehicle vs. 10 µg/mL *M. vaccae* NCTC 11659 + 0.5 ng/mL LPS	> .1
			10 µg/mL *M. vaccae* NCTC 11659 + LPS vehicle vs. 10 µg/mL *M. vaccae* NCTC 11659 + 2.5 ng/mL LPS	> .1
			10 µg/mL *M. vaccae* NCTC 11659 + LPS vehicle vs. 10 µg/mL *M. vaccae* NCTC 11659 + 250 ng/mL LPS	0.02535678
		Between all LPS conditions within 30 µg/mL *M. vaccae* NCTC 11659	30 µg/mL *M. vaccae* NCTC 11659 + LPS vehicle vs. 30 µg/mL *M. vaccae* NCTC 11659 + 0.5 ng/mL LPS	0.0625251
			30 µg/mL *M. vaccae* NCTC 11659 + LPS vehicle vs. 30 µg/mL *M. vaccae* NCTC 11659 + 2.5 ng/mL LPS	> .1
			30 µg/mL *M. vaccae* NCTC 11659 + LPS vehicle vs. 30 µg/mL *M. vaccae* NCTC 11659 + 250 ng/mL LPS	> .1

			100 µg/mL *M. vaccae* NCTC 11659 + LPS vehicle vs. 100 µg/mL *M. vaccae* NCTC 11659 + 0.5 ng/mL LPS	> .1
		Between all LPS conditions within 100 µg/mL *M. vaccae* NCTC 11659	100 µg/mL *M. vaccae* NCTC 11659 + LPS vehicle vs. 100 µg/mL *M. vaccae* NCTC 11659 + 2.5 ng/mL LPS	> .1
			100 µg/mL *M. vaccae* NCTC 11659 + LPS vehicle vs. 100 µg/mL *M. vaccae* NCTC 11659 + 250 ng/mL LPS	> .1
			300 µg/mL *M. vaccae* NCTC 11659 + LPS vehicle vs. 300 µg/mL *M. vaccae* NCTC 11659 + 0.5 ng/mL LPS	> .1
		Between all LPS conditions within 300 µg/mL *M. vaccae* NCTC 11659	300 µg/mL *M. vaccae* NCTC 11659 + LPS vehicle vs. 300 µg/mL *M. vaccae* NCTC 11659 + 2.5 ng/mL LPS	> .1
			300 µg/mL *M. vaccae* NCTC 11659 + LPS vehicle vs. 300 µg/mL *M. vaccae* NCTC 11659 + 250 ng/mL LPS	0.01371849
	IL10:IL12B		BBS vs. 10 µg/mL *M. vaccae* NCTC 11659	> .1
		Within 0 ng/mL	BBS vs. 30 µg/mL *M. vaccae* NCTC 11659	< .01
			BBS vs. 100 µg/mL *M. vaccae* NCTC 11659	< .01
			BBS vs. 300 µg/mL *M. vaccae* NCTC 11659	< .001
			BBS vs. 10 µg/mL *M. vaccae* NCTC 11659	> .1
		Within 0.5 ng/mL	BBS vs. 30 µg/mL *M. vaccae* NCTC 11659	> .1
			BBS vs. 100 µg/mL *M. vaccae* NCTC 11659	< .01
			BBS vs. 300 µg/mL *M. vaccae* NCTC 11659	< .001
			BBS vs. 10 µg/mL *M. vaccae* NCTC 11659	0.03528282
		Within 2.5 ng/mL	BBS vs. 30 µg/mL *M. vaccae* NCTC 11659	0.01126892
			BBS vs. 100 µg/mL *M. vaccae* NCTC 11659	> .1

			BBS vs. 300 µg/mL *M. vaccae* NCTC 11659	> .1
		Within 250 ng/mL	BBS vs. 10 µg/mL *M. vaccae* NCTC 11659	> .1
			BBS vs. 30 µg/mL *M. vaccae* NCTC 11659	0.06619748
			BBS vs. 100 µg/mL *M. vaccae* NCTC 11659	< .01
			BBS vs. 300 µg/mL *M. vaccae* NCTC 11659	< .01
		Between all LPS conditions within BBS	BBS + LPS vehicle vs. BBS + 0.5 ng/mL LPS	0.04940273
			BBS + LPS vehicle vs. BBS + 2.5 ng/mL LPS	< .001
			BBS + LPS vehicle vs. BBS + 250 ng/mL LPS	< .001
		Between all LPS conditions within 10 µg/mL *M. vaccae* NCTC 11659	10 µg/mL *M. vaccae* NCTC 11659 + LPS vehicle vs. 10 µg/mL *M. vaccae* NCTC 11659 + 0.5 ng/mL LPS	0.04720208
			10 µg/mL *M. vaccae* NCTC 11659 + LPS vehicle vs. 10 µg/mL *M. vaccae* NCTC 11659 + 2.5 ng/mL LPS	< .01
			10 µg/mL *M. vaccae* NCTC 11659 + LPS vehicle vs. 10 µg/mL *M. vaccae* NCTC 11659 + 250 ng/mL LPS	< .001
		Between all LPS conditions within 30 µg/mL *M. vaccae* NCTC 11659	30 µg/mL *M. vaccae* NCTC 11659 + LPS vehicle vs. 30 µg/mL *M. vaccae* NCTC 11659 + 0.5 ng/mL LPS	> .1
			30 µg/mL *M. vaccae* NCTC 11659 + LPS vehicle vs. 30 µg/mL *M. vaccae* NCTC 11659 + 2.5 ng/mL LPS	> .1
			30 µg/mL *M. vaccae* NCTC 11659 + LPS vehicle vs. 30 µg/mL *M. vaccae* NCTC 11659 + 250 ng/mL LPS	< .001
		Between all LPS conditions within 100 µg/mL *M. vaccae* NCTC 11659	100 µg/mL *M. vaccae* NCTC 11659 + LPS vehicle vs. 100 µg/mL *M. vaccae* NCTC 11659 + 0.5 ng/mL LPS	> .1

			100 µg/mL *M. vaccae* NCTC 11659 + LPS vehicle vs. 100 µg/mL *M. vaccae* NCTC 11659 + 2.5 ng/mL LPS	> .1
			100 µg/mL *M. vaccae* NCTC 11659 + LPS vehicle vs. 100 µg/mL *M. vaccae* NCTC 11659 + 250 ng/mL LPS	< .001
		Between all LPS conditions within 300 µg/mL *M. vaccae* NCTC 11659	300 µg/mL *M. vaccae* NCTC 11659 + LPS vehicle vs. 300 µg/mL *M. vaccae* NCTC 11659 + 0.5 ng/mL LPS	> .1
			300 µg/mL *M. vaccae* NCTC 11659 + LPS vehicle vs. 300 µg/mL *M. vaccae* NCTC 11659 + 2.5 ng/mL LPS	> .1
			300 µg/mL *M. vaccae* NCTC 11659 + LPS vehicle vs. 300 µg/mL *M. vaccae* NCTC 11659 + 250 ng/mL LPS	< .001
	IL10:IL23A	Within 0 ng/mL	BBS vs. 10 µg/mL *M. vaccae* NCTC 11659	> .1
			BBS vs. 30 µg/mL *M. vaccae* NCTC 11659	0.01920024
			BBS vs. 100 µg/mL *M. vaccae* NCTC 11659	< .01
			BBS vs. 300 µg/mL *M. vaccae* NCTC 11659	< .001
		Within 0.5 ng/mL	BBS vs. 10 µg/mL *M. vaccae* NCTC 11659	> .1
			BBS vs. 30 µg/mL *M. vaccae* NCTC 11659	> .1
			BBS vs. 100 µg/mL *M. vaccae* NCTC 11659	< .01
			BBS vs. 300 µg/mL *M. vaccae* NCTC 11659	< .01
		Within 2.5 ng/mL	BBS vs. 10 µg/mL *M. vaccae* NCTC 11659	> .1
			BBS vs. 30 µg/mL *M. vaccae* NCTC 11659	> .1
			BBS vs. 100 µg/mL *M. vaccae* NCTC 11659	> .1
			BBS vs. 300 µg/mL *M. vaccae* NCTC 11659	0.01299137
		Within 250 ng/mL	BBS vs. 10 µg/mL *M. vaccae* NCTC 11659	> .1

			BBS vs. 30 µg/mL *M. vaccae* NCTC 11659	0.01408542
			BBS vs. 100 µg/mL *M. vaccae* NCTC 11659	< .01
			BBS vs. 300 µg/mL *M. vaccae* NCTC 11659	0.01154322
		Between all LPS conditions within BBS	BBS + LPS vehicle vs. BBS + 0.5 ng/mL LPS	0.04855252
			BBS + LPS vehicle vs. BBS + 2.5 ng/mL LPS	< .01
			BBS + LPS vehicle vs. BBS + 250 ng/mL LPS	< .001
		Between all LPS conditions within 10 µg/mL *M. vaccae* NCTC 11659	10 µg/mL *M. vaccae* NCTC 11659 + LPS vehicle vs. 10 µg/mL *M. vaccae* NCTC 11659 + 0.5 ng/mL LPS	> .1
			10 µg/mL *M. vaccae* NCTC 11659 + LPS vehicle vs. 10 µg/mL *M. vaccae* NCTC 11659 + 2.5 ng/mL LPS	< .01
			10 µg/mL *M. vaccae* NCTC 11659 + LPS vehicle vs. 10 µg/mL *M. vaccae* NCTC 11659 + 250 ng/mL LPS	< .001
		Between all LPS conditions within 30 µg/mL *M. vaccae* NCTC 11659	30 µg/mL *M. vaccae* NCTC 11659 + LPS vehicle vs. 30 µg/mL *M. vaccae* NCTC 11659 + 0.5 ng/mL LPS	> .1
			30 µg/mL *M. vaccae* NCTC 11659 + LPS vehicle vs. 30 µg/mL *M. vaccae* NCTC 11659 + 2.5 ng/mL LPS	> .1
			30 µg/mL *M. vaccae* NCTC 11659 + LPS vehicle vs. 30 µg/mL *M. vaccae* NCTC 11659 + 250 ng/mL LPS	< .001
		Between all LPS conditions within 100 µg/mL *M. vaccae* NCTC 11659	100 µg/mL *M. vaccae* NCTC 11659 + LPS vehicle vs. 100 µg/mL *M. vaccae* NCTC 11659 + 0.5 ng/mL LPS	> .1
			100 µg/mL *M. vaccae* NCTC 11659 + LPS vehicle vs. 100 µg/mL *M. vaccae* NCTC 11659 + 2.5 ng/mL LPS	0.07592508

			100 µg/mL *M. vaccae* NCTC 11659 + LPS vehicle vs. 100 µg/mL *M. vaccae* NCTC 11659 + 250 ng/mL LPS	< .001
		Between all LPS conditions within 300 µg/mL *M. vaccae* NCTC 11659	300 µg/mL *M. vaccae* NCTC 11659 + LPS vehicle vs. 300 µg/mL *M. vaccae* NCTC 11659 + 0.5 ng/mL LPS	> .1
			300 µg/mL *M. vaccae* NCTC 11659 + LPS vehicle vs. 300 µg/mL *M. vaccae* NCTC 11659 + 2.5 ng/mL LPS	> .1
			300 µg/mL *M. vaccae* NCTC 11659 + LPS vehicle vs. 300 µg/mL *M. vaccae* NCTC 11659 + 250 ng/mL LPS	< .001
S15	*TGFB:IL12A*	Within 0 ng/mL	BBS vs. 10 µg/mL *M. vaccae* NCTC 11659	> .1
			BBS vs. 30 µg/mL *M. vaccae* NCTC 11659	> .1
			BBS vs. 100 µg/mL *M. vaccae* NCTC 11659	> .1
			BBS vs. 300 µg/mL *M. vaccae* NCTC 11659	> .1
		Within 0.5 ng/mL	BBS vs. 10 µg/mL *M. vaccae* NCTC 11659	> .1
			BBS vs. 30 µg/mL *M. vaccae* NCTC 11659	> .1
			BBS vs. 100 µg/mL *M. vaccae* NCTC 11659	> .1
			BBS vs. 300 µg/mL *M. vaccae* NCTC 11659	> .1
		Within 2.5 ng/mL	BBS vs. 10 µg/mL *M. vaccae* NCTC 11659	> .1
			BBS vs. 30 µg/mL *M. vaccae* NCTC 11659	0.07904917
			BBS vs. 100 µg/mL *M. vaccae* NCTC 11659	0.09003819
			BBS vs. 300 µg/mL *M. vaccae* NCTC 11659	< .01
		Within 250 ng/mL	BBS vs. 10 µg/mL *M. vaccae* NCTC 11659	> .1
			BBS vs. 30 µg/mL *M. vaccae* NCTC 11659	> .1
			BBS vs. 100 µg/mL *M. vaccae* NCTC 11659	> .1

			BBS vs. 300 µg/mL *M. vaccae* NCTC 11659	> .1
		Between all LPS conditions within BBS	BBS + LPS vehicle vs. BBS + 0.5 ng/mL LPS	> .1
			BBS + LPS vehicle vs. BBS + 2.5 ng/mL LPS	> .1
			BBS + LPS vehicle vs. BBS + 250 ng/mL LPS	> .1
		Between all LPS conditions within 10 µg/mL *M. vaccae* NCTC 11659	10 µg/mL *M. vaccae* NCTC 11659 + LPS vehicle vs. 10 µg/mL *M. vaccae* NCTC 11659 + 0.5 ng/mL LPS	> .1
			10 µg/mL *M. vaccae* NCTC 11659 + LPS vehicle vs. 10 µg/mL *M. vaccae* NCTC 11659 + 2.5 ng/mL LPS	> .1
			10 µg/mL *M. vaccae* NCTC 11659 + LPS vehicle vs. 10 µg/mL *M. vaccae* NCTC 11659 + 250 ng/mL LPS	0.02780981
		Between all LPS conditions within 30 µg/mL *M. vaccae* NCTC 11659	30 µg/mL *M. vaccae* NCTC 11659 + LPS vehicle vs. 30 µg/mL *M. vaccae* NCTC 11659 + 0.5 ng/mL LPS	> .1
			30 µg/mL *M. vaccae* NCTC 11659 + LPS vehicle vs. 30 µg/mL *M. vaccae* NCTC 11659 + 2.5 ng/mL LPS	< .01
			30 µg/mL *M. vaccae* NCTC 11659 + LPS vehicle vs. 30 µg/mL *M. vaccae* NCTC 11659 + 250 ng/mL LPS	> .1
		Between all LPS conditions within 100 µg/mL *M. vaccae* NCTC 11659	100 µg/mL *M. vaccae* NCTC 11659 + LPS vehicle vs. 100 µg/mL *M. vaccae* NCTC 11659 + 0.5 ng/mL LPS	> .1
			100 µg/mL *M. vaccae* NCTC 11659 + LPS vehicle vs. 100 µg/mL *M. vaccae* NCTC 11659 + 2.5 ng/mL LPS	> .1
			100 µg/mL *M. vaccae* NCTC 11659 + LPS vehicle vs. 100 µg/mL *M. vaccae* NCTC 11659 + 250 ng/mL LPS	> .1

		Between all LPS conditions within 300 µg/mL *M. vaccae* NCTC 11659	300 µg/mL *M. vaccae* NCTC 11659 + LPS vehicle vs. 300 µg/mL *M. vaccae* NCTC 11659 + 0.5 ng/mL LPS	> .1
			300 µg/mL *M. vaccae* NCTC 11659 + LPS vehicle vs. 300 µg/mL *M. vaccae* NCTC 11659 + 2.5 ng/mL LPS	> .1
			300 µg/mL *M. vaccae* NCTC 11659 + LPS vehicle vs. 300 µg/mL *M. vaccae* NCTC 11659 + 250 ng/mL LPS	> .1
	TGFB:IL12B	Within 0 ng/mL	BBS vs. 10 µg/mL *M. vaccae* NCTC 11659	> .1
			BBS vs. 30 µg/mL *M. vaccae* NCTC 11659	0.01007759
			BBS vs. 100 µg/mL *M. vaccae* NCTC 11659	< .01
			BBS vs. 300 µg/mL *M. vaccae* NCTC 11659	< .01
		Within 0.5 ng/mL	BBS vs. 10 µg/mL *M. vaccae* NCTC 11659	> .1
			BBS vs. 30 µg/mL *M. vaccae* NCTC 11659	> .1
			BBS vs. 100 µg/mL *M. vaccae* NCTC 11659	0.08479317
			BBS vs. 300 µg/mL *M. vaccae* NCTC 11659	0.01243819
		Within 2.5 ng/mL	BBS vs. 10 µg/mL *M. vaccae* NCTC 11659	> .1
			BBS vs. 30 µg/mL *M. vaccae* NCTC 11659	> .1
			BBS vs. 100 µg/mL *M. vaccae* NCTC 11659	0.01583241
			BBS vs. 300 µg/mL *M. vaccae* NCTC 11659	0.0236974
		Within 250 ng/mL	BBS vs. 10 µg/mL *M. vaccae* NCTC 11659	> .1
			BBS vs. 30 µg/mL *M. vaccae* NCTC 11659	> .1
			BBS vs. 100 µg/mL *M. vaccae* NCTC 11659	> .1
			BBS vs. 300 µg/mL *M. vaccae* NCTC 11659	< .01
			BBS + LPS vehicle vs. BBS + 0.5 ng/mL LPS	0.01706393

		Between all LPS conditions within BBS	BBS + LPS vehicle vs. BBS + 2.5 ng/mL LPS	< .01
			BBS + LPS vehicle vs. BBS + 250 ng/mL LPS	< .001
		Between all LPS conditions within 10 µg/mL *M. vaccae* NCTC 11659	10 µg/mL *M. vaccae* NCTC 11659 + LPS vehicle vs. 10 µg/mL *M. vaccae* NCTC 11659 + 0.5 ng/mL LPS	> .1
			10 µg/mL *M. vaccae* NCTC 11659 + LPS vehicle vs. 10 µg/mL *M. vaccae* NCTC 11659 + 2.5 ng/mL LPS	< .01
			10 µg/mL *M. vaccae* NCTC 11659 + LPS vehicle vs. 10 µg/mL *M. vaccae* NCTC 11659 + 250 ng/mL LPS	< .001
		Between all LPS conditions within 30 µg/mL *M. vaccae* NCTC 11659	30 µg/mL *M. vaccae* NCTC 11659 + LPS vehicle vs. 30 µg/mL *M. vaccae* NCTC 11659 + 0.5 ng/mL LPS	> .1
			30 µg/mL *M. vaccae* NCTC 11659 + LPS vehicle vs. 30 µg/mL *M. vaccae* NCTC 11659 + 2.5 ng/mL LPS	< .01
			30 µg/mL *M. vaccae* NCTC 11659 + LPS vehicle vs. 30 µg/mL *M. vaccae* NCTC 11659 + 250 ng/mL LPS	< .001
		Between all LPS conditions within 100 µg/mL *M. vaccae* NCTC 11659	100 µg/mL *M. vaccae* NCTC 11659 + LPS vehicle vs. 100 µg/mL *M. vaccae* NCTC 11659 + 0.5 ng/mL LPS	> .1
			100 µg/mL *M. vaccae* NCTC 11659 + LPS vehicle vs. 100 µg/mL *M. vaccae* NCTC 11659 + 2.5 ng/mL LPS	> .1
			100 µg/mL *M. vaccae* NCTC 11659 + LPS vehicle vs. 100 µg/mL *M. vaccae* NCTC 11659 + 250 ng/mL LPS	< .001
		Between all LPS conditions within 300 µg/mL *M. vaccae* NCTC 11659	300 µg/mL *M. vaccae* NCTC 11659 + LPS vehicle vs. 300 µg/mL *M. vaccae* NCTC 11659 + 0.5 ng/mL LPS	> .1

			300 µg/mL *M. vaccae* NCTC 11659 + LPS vehicle vs. 300 µg/mL *M. vaccae* NCTC 11659 + 2.5 ng/mL LPS	> .1
			300 µg/mL *M. vaccae* NCTC 11659 + LPS vehicle vs. 300 µg/mL *M. vaccae* NCTC 11659 + 250 ng/mL LPS	< .01
	TGFB:IL23A	Within 0 ng/mL	BBS vs. 10 µg/mL *M. vaccae* NCTC 11659	> .1
			BBS vs. 30 µg/mL *M. vaccae* NCTC 11659	> .1
			BBS vs. 100 µg/mL *M. vaccae* NCTC 11659	< .01
			BBS vs. 300 µg/mL *M. vaccae* NCTC 11659	< .01
		Within 0.5 ng/mL	BBS vs. 10 µg/mL *M. vaccae* NCTC 11659	> .1
			BBS vs. 30 µg/mL *M. vaccae* NCTC 11659	> .1
			BBS vs. 100 µg/mL *M. vaccae* NCTC 11659	> .1
			BBS vs. 300 µg/mL *M. vaccae* NCTC 11659	> .1
		Within 2.5 ng/mL	BBS vs. 10 µg/mL *M. vaccae* NCTC 11659	> .1
			BBS vs. 30 µg/mL *M. vaccae* NCTC 11659	> .1
			BBS vs. 100 µg/mL *M. vaccae* NCTC 11659	0.02776241
			BBS vs. 300 µg/mL *M. vaccae* NCTC 11659	0.03448704
		Within 250 ng/mL	BBS vs. 10 µg/mL *M. vaccae* NCTC 11659	> .1
			BBS vs. 30 µg/mL *M. vaccae* NCTC 11659	> .1
			BBS vs. 100 µg/mL *M. vaccae* NCTC 11659	0.0724892
			BBS vs. 300 µg/mL *M. vaccae* NCTC 11659	< .01
		Between all LPS conditions within BBS	BBS + LPS vehicle vs. BBS + 0.5 ng/mL LPS	< .01
			BBS + LPS vehicle vs. BBS + 2.5 ng/mL LPS	< .001
			BBS + LPS vehicle vs. BBS + 250 ng/mL LPS	< .001

		Between all LPS conditions within 10 µg/mL *M. vaccae* NCTC 11659	10 µg/mL *M. vaccae* NCTC 11659 + LPS vehicle vs. 10 µg/mL *M. vaccae* NCTC 11659 + 0.5 ng/mL LPS	> .1
			10 µg/mL *M. vaccae* NCTC 11659 + LPS vehicle vs. 10 µg/mL *M. vaccae* NCTC 11659 + 2.5 ng/mL LPS	< .01
			10 µg/mL *M. vaccae* NCTC 11659 + LPS vehicle vs. 10 µg/mL *M. vaccae* NCTC 11659 + 250 ng/mL LPS	< .001
		Between all LPS conditions within 30 µg/mL *M. vaccae* NCTC 11659	30 µg/mL *M. vaccae* NCTC 11659 + LPS vehicle vs. 30 µg/mL *M. vaccae* NCTC 11659 + 0.5 ng/mL LPS	> .1
			30 µg/mL *M. vaccae* NCTC 11659 + LPS vehicle vs. 30 µg/mL *M. vaccae* NCTC 11659 + 2.5 ng/mL LPS	< .001
			30 µg/mL *M. vaccae* NCTC 11659 + LPS vehicle vs. 30 µg/mL *M. vaccae* NCTC 11659 + 250 ng/mL LPS	< .001
		Between all LPS conditions within 100 µg/mL *M. vaccae* NCTC 11659	100 µg/mL *M. vaccae* NCTC 11659 + LPS vehicle vs. 100 µg/mL *M. vaccae* NCTC 11659 + 0.5 ng/mL LPS	> .1
			100 µg/mL *M. vaccae* NCTC 11659 + LPS vehicle vs. 100 µg/mL *M. vaccae* NCTC 11659 + 2.5 ng/mL LPS	> .1
			100 µg/mL *M. vaccae* NCTC 11659 + LPS vehicle vs. 100 µg/mL *M. vaccae* NCTC 11659 + 250 ng/mL LPS	< .01
		Between all LPS conditions within 300 µg/mL *M. vaccae* NCTC 11659	300 µg/mL *M. vaccae* NCTC 11659 + LPS vehicle vs. 300 µg/mL *M. vaccae* NCTC 11659 + 0.5 ng/mL LPS	> .1
			300 µg/mL *M. vaccae* NCTC 11659 + LPS vehicle vs. 300 µg/mL *M. vaccae* NCTC 11659 + 2.5 ng/mL LPS	> .1

			300 µg/mL *M. vaccae* NCTC 11659 + LPS vehicle vs. 300 µg/mL *M. vaccae* NCTC 11659 + 250 ng/mL LPS	< .01

Table S11. Primers used for real time RT-PCR listed from 5' to 3'.

Gene	Forward Primer	Reverse Primer	Sequence Name
ACTB	GGACCTGACTGACTACCTCAT	CGTAGCACAGCTTCTCCTTAAT	ACC# NM_001101.3
IL10	GCTGGAGGACTTTAAGGGTTAC	GATGTCTGGGTCTTGGTTCTC	ACC# NM_000572.3
IL12A	CCAGAAGAATGAGAGTTGCCTAA	GGCACAGGGCCATCATAAA	ACC# NM_001354583.2
IL12B	CACATTCCTACTTCTCCCTGAC	GTCCGTGAAGACTCTATCTTTCTT	ACC# AF180563.1
IL23A	AGAGAAGAGGGAGATGAAGAGA	CCTTTGCAAGCAGAACTGAC	ACC# NM_016584.3
TGFB1	GTAAGAAGCACGAGCTGTATGT	GCGTTCATGTAGGAGTTCAGAG	ACC# M60316.1

S4. Supplemental References

References

Grohmann, U.; Belladonna, M.L.; Vacca, C.; Bianchi, R.; Fallarino, F.; Orabona, C.; Fioretti, M.C.; Puccetti, P. Positive regulatory role of IL-12 in macrophages and modulation by IFN-gamma. *J. Immunol.* **2001**, *167*, 221–227. https://doi.org/10.4049/jimmunol.167.1.221.

Ma, X.; Chow, J.M.; Gri, G.; Carra, G.; Gerosa, F.; Wolf, S.F.; Dzialo, R.; Trinchieri, G. The interleukin 12 p40 gene promoter is primed by interferon gamma in monocytic cells. *J. Exp. Med.* **1996**, *183*, 147–157. https://doi.org/10.1084/jem.183.1.147.

Ma, X.; Yan, W.; Zheng, H.; Du, Q.; Zhang, L.; Ban, Y.; Li, N.; Wei, F. Regulation of IL-10 and IL-12 production and function in macrophages and dendritic cells. *F1000Res* **2015**, *4*. https://doi.org/10.12688/f1000research.7010.1.

Mantovani, A.; Sica, A.; Sozzani, S.; Allavena, P.; Vecchi, A.; Locati, M. The chemokine system in diverse forms of macrophage activation and polarization. *Trends Immunol.* **2004**, *25*, 677–686. https://doi.org/10.1016/j.it.2004.09.015.

Viola, A.; Munari, F.; Sánchez-Rodríguez, R.; Scolaro, T.; Castegna, A. The metabolic signature of macrophage responses. *Front. Immunol.* **2019**, *10*, 1462. https://doi.org/10.3389/fimmu.2019.01462.
